# Pathophysiology of LV Remodeling in Survivors of STEMI: Inflammation, Remote Myocardium, and Prognosis

**DOI:** 10.1016/j.jcmg.2015.03.007

**Published:** 2015-07

**Authors:** David Carrick, Caroline Haig, Sam Rauhalammi, Nadeem Ahmed, Ify Mordi, Margaret McEntegart, Mark C. Petrie, Hany Eteiba, Mitchell Lindsay, Stuart Watkins, Stuart Hood, Andrew Davie, Ahmed Mahrous, Naveed Sattar, Paul Welsh, Niko Tzemos, Aleksandra Radjenovic, Ian Ford, Keith G. Oldroyd, Colin Berry

**Affiliations:** ∗ BHF Glasgow Cardiovascular Research Centre, Institute of Cardiovascular and Medical Sciences, University of Glasgow, Glasgow, United Kingdom; † West of Scotland Heart and Lung Centre, Golden Jubilee National Hospital, Glasgow, United Kingdom; ‡ Robertson Centre for Biostatistics, University of Glasgow, Glasgow, United Kingdom

**Keywords:** cardiac magnetic resonance, inflammation, myocardial infarction, remodeling, reperfusion, CMR, cardiac magnetic resonance, CRP, C-reactive protein, ECG, electrocardiogram, LV, left ventricular, MACE, major adverse cardiac events, NT-proBNP, N-terminal pro–B-type natriuretic peptide, PCI, percutaneous coronary intervention, STEMI, ST-segment elevation myocardial infarction

## Abstract

**Objectives:**

The aim of this study was to investigate the clinical significance of native T1 values in remote myocardium in survivors of acute ST-segment elevation myocardial infarction (STEMI).

**Background:**

The pathophysiology and prognostic significance of remote myocardium in the natural history of STEMI is uncertain. Cardiac magnetic resonance (CMR) reveals myocardial function and pathology. Native T1 (relaxation time in ms) is a fundamental magnetic resonance tissue property determined by water content and cellularity.

**Results:**

A total of 300 STEMI patients (mean age 59 years; 74% male) gave informed consent. A total of 288 STEMI patients had evaluable native T1 CMR, and 267 patients (91%) had follow-up CMR at 6 months. Health outcome information was obtained for all of the participants (median follow-up 845 days). Infarct size was 18 ± 13% of left ventricular (LV) mass. Two days post-STEMI, native T1 was lower in remote myocardium than in the infarct zone (961 ± 25 ms vs. 1,097 ± 52 ms; p < 0.01). In multivariable regression, incomplete ST-segment resolution was associated with myocardial remote zone native T1 (regression coefficient 9.42; 95% confidence interval [CI]: 2.37 to 16.47; p = 0.009), as were the log of the admission C-reactive protein concentration (3.01; 95% CI: 0.016 to 5.85; p = 0.038) and the peak monocyte count (10.20; 95% CI: 0.74 to 19.67; p = 0.035). Remote T1 at baseline was associated with log N-terminal pro–B-type natriuretic peptide at 6 months (0.01; 95% CI: 0.00 to 0.02; p = 0.002; n = 151) and the change in LV end-diastolic volume from baseline to 6 months (0.13; 95% CI: 0.01 to 0.24; p = 0.035). Remote zone native T1 was independently associated with post-discharge major adverse cardiac events (n = 20 events; hazard ratio: 1.016; 95% CI: 1.000 to 1.032; p = 0.048) and all-cause death or heart failure hospitalization (n = 30 events during admission and post-discharge; hazard ratio: 1.014; 95% CI: 1.000 to 1.028; p = 0.049).

**Conclusions:**

Reperfusion injury and inflammation early post-MI was associated with remote zone T1, which in turn was independently associated with LV remodeling and adverse cardiac events post-STEMI. (Detection and Significance of Heart Injury in ST Elevation Myocardial Infarction [BHF MR-MI]; NCT02072850)

The pathophysiology of left ventricular (LV) dysfunction and remodeling after acute ST-segment elevation myocardial infarction (STEMI) is incompletely understood 1, 2, 3, 4. Acute MI triggers a systemic acute-phase response, and neutrophils and monocytes/macrophages track to infarct 5, 6, 7 and remote 8, 9 myocardial tissues from reticuloendothelial stores 5, 6, 7. Proof-of-concept studies in humans support the experimental observations 8, 10. Local cytokine production from cardiomyocytes and macrophages represents an acute stress response to injury within the first week post-MI 9, 11, leading to maladaptive matrix modifications that are associated with impaired contractility in the myocardial remote zone (7). Systemic inflammation is prognostically important post-MI (12), and evidence-based therapies for MI may reduce inflammatory activation (13).

Human tissue has fundamental magnetic properties, including the longitudinal (spin-lattice) proton relaxation time (native T1 in ms). Native T1 is influenced by water content, binding with macromolecules, and cell composition 14, 15. Tissue water content increases as a result of ischemia, resulting in longer T1 times being a biomarker of more severe myocardial injury in localized myocardial regions 15, 16, 17. Cardiac magnetic resonance (CMR) now enables spatially resolved measurement of native T1 (T1 mapping) in the heart without using a gadolinium contrast agent that is normally required to delineate infarct tissue. However, the potential of native T1 to be used as a novel biomarker of heart injury in STEMI patients is not completely understood 7, 18, 19, 20, 21, 22, 23, 24 (Figure 1).

**Figure 1:**
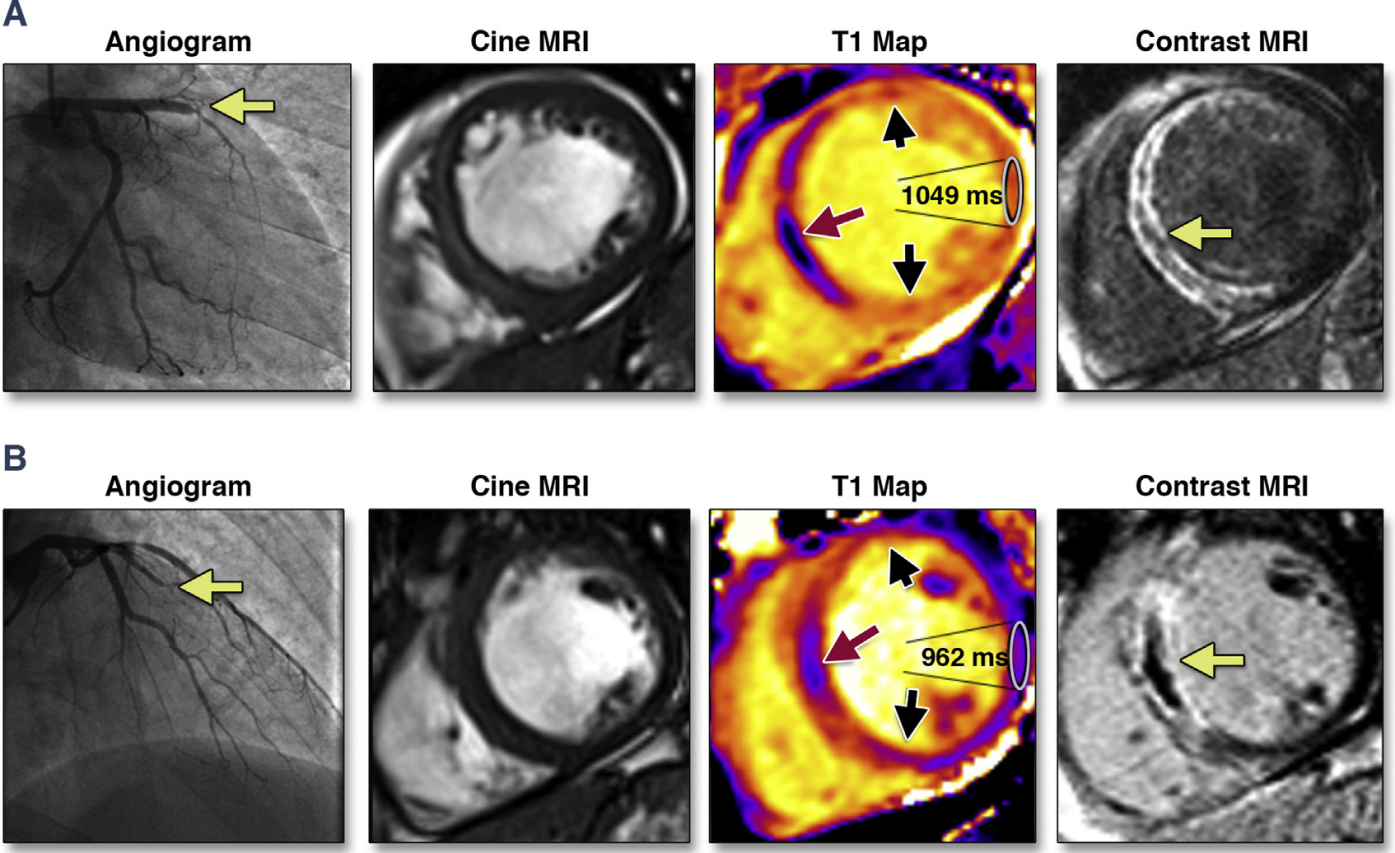
Two Patients With Anterior STEMI and Divergent Clinical Courses Both patients had similar clinical presentations, with acute anterior ST-segment elevation myocardial infarction (STEMI) treated by primary percutaneous coronary intervention. The yellow arrow indicates the location of the thrombotic occlusion of the **(A)** left anterior descending, and **(B)** obtuse marginal, branch of the left coronary artery. The history of each patient is described in the Online Appendix. MRI = magnetic resonance imaging.

We hypothesized that regional myocardial tissue characteristics reflected by native T1 would be associated with the initial extent of ischemic injury and systemic inflammation. Our second hypothesis was that remote myocardial characteristics (native T1) would be independently associated with LV remodeling and pre-defined cardiovascular outcomes and mortality in the longer term.

## Methods

### Study population and STEMI management

We performed a prospective CMR cohort study in a single regional cardiac center between May 11, 2011, and November 22, 2012. A total of 343 STEMI patients provided written informed consent. The eligibility criteria included an indication for primary percutaneous coronary intervention (PCI) or thrombolysis for STEMI (25). Exclusion criteria represented standard contraindications to contrast CMR. Acute STEMI management followed contemporary guidelines 25, 26. The study had ethics approval (reference 10-S0703-28) and was publically registered. Fifty healthy volunteers also underwent CMR (Online Appendix).

### CMR acquisition

CMR was performed on a MAGNETOM Avanto (Siemens Healthcare, Erlangen, Germany) 1.5-T scanner with a 12-element phased-array cardiac surface coil 2 days and 6 months post-MI 27, 28. The imaging protocol (Online Appendix) included cine CMR with steady-state free precession, native T1 relaxometry (mapping) using an optimized modified look-locker inversion-recovery investigational prototype sequence (work-in-progress 448, Siemens Healthcare) 21, 22, 29, T2 mapping 30, 31, and delayed-enhancement phase-sensitive inversion-recovery pulse sequences (32). Patients and healthy volunteers underwent the same imaging protocol, with the exception that healthy volunteers <45 years of age did not receive gadolinium (Online Appendix).

### CMR analyses

The CMR analyses are described in the Online Appendix.

#### T1-standardized measurements in myocardial regions of interest

LV contours were delineated with computer-assisted planimetry on the raw T1 image and copied onto the color-encoded spatially coregistered maps. Apical segments were not included because of partial volume effects. Particular care was taken to delineate regions of interest with adequate margins of separation from tissue interfaces prone to partial volume averaging, such as between myocardium and blood 27, 28, 33. The presence of off-resonance artifacts and cardiorespiratory motion was assessed by examination of the raw T1-weighted images. A total of 300 segments (8.6%) were excluded from analysis due to the presence of off-resonance or motion artifacts.

In STEMI patients, myocardial T1 values were segmented spatially and regions of interest were defined as: 1) remote myocardium; 2) injured myocardium; or 3) infarct core. The regions of interest were planimetered to include the entire area of interest with distinct margins of separation from tissue interfaces to exclude partial volume averaging. The remote myocardial region of interest was defined as myocardium 180° from the affected zone with no visible evidence of infarction, edema, or wall motion abnormalities (assessed by inspecting corresponding contrast-enhanced T1-weighted, T2-weighted, or cine images, respectively). The infarct zone region of interest was defined as myocardium with pixel values (T1 or T2) >2 SD from remote myocardium on T2-weighted CMR 29, 30, 31. The infarct core was defined as an area in the center of the infarct territory having a mean T1 value of at least 2 SD below the T1 value of the periphery of the area at risk.

#### Infarct definition and size

The myocardial mass of late gadolinium (g) was quantified using computer-assisted planimetry, and the territory of infarction was delineated using a signal intensity threshold of >5 SD above a remote reference region and expressed as a percentage of total LV mass (33).

#### Area at risk

Area at risk was defined as LV myocardium with pixel values (T1/T2) >2 SD from remote myocardium 4, 23, 24, 34, 35, 36.

#### Myocardial salvage

Myocardial salvage was calculated by subtraction of percentage infarct size from percentage area at risk 4, 34, 35, 36. The myocardial salvage index was calculated by dividing the myocardial salvage area by the initial area at risk.

#### Adverse remodeling

Adverse remodeling was defined as an increase in LV end-diastolic volume ≥20% at 6 months from baseline (3).

### Laboratory analyses

The acquisition of the electrocardiograms (ECGs) and blood samples for biochemical and hematologic analyses are described in the Online Appendix.

### Pre-specified health outcomes

We pre-specified adverse health outcomes that are pathophysiologically linked with STEMI. The primary composite outcome was major adverse cardiac events (MACE), defined as cardiac death, nonfatal MI, or hospitalization for heart failure (Online Appendix). All-cause death or heart failure hospitalization was a secondary outcome. The serious adverse events were independently assessed by a cardiologist who was not a member of the research team. The serious adverse events were defined according to standard guidelines (Online Appendix) and categorized as having occurred either during the index admission or post-discharge. All study participants were followed up by patient contacts through telephone calls, clinic visits, and review of the electronic medical records for a minimum of 18 months after discharge.

### Statistical analyses

The sample size calculation and statistical methods are described in the Online Appendix. Myocardial native T1 values in spatially matched regions of the heart (37) in STEMI patients and healthy volunteers were compared using independent 2-sample Student *t* tests. Univariable and multivariable linear regression analyses were performed to identify associations of T1 values for: 1) remote myocardium; 2) injured myocardium; and 3) infarct core in all patients and in patients without late microvascular obstruction (3). Binary logistic regression models were used to identify associates of adverse remodeling at the 6-month follow-up. In stepwise linear regressions, the Akaike information criterion (AIC) was used as a measure of the relative quality of the models for this dataset, and the model with the minimum AIC value was reported. Kaplan-Meier and Cox proportional hazards methods were used to identify potential clinical correlates of MACE and all-cause death/heart failure events, including patient characteristics, CMR findings, and native T1. A p value >0.05 indicated the absence of a statistically significant effect. The natural log has been used in any logarithmic transforms of variables.

## Results

**Figure 2:**
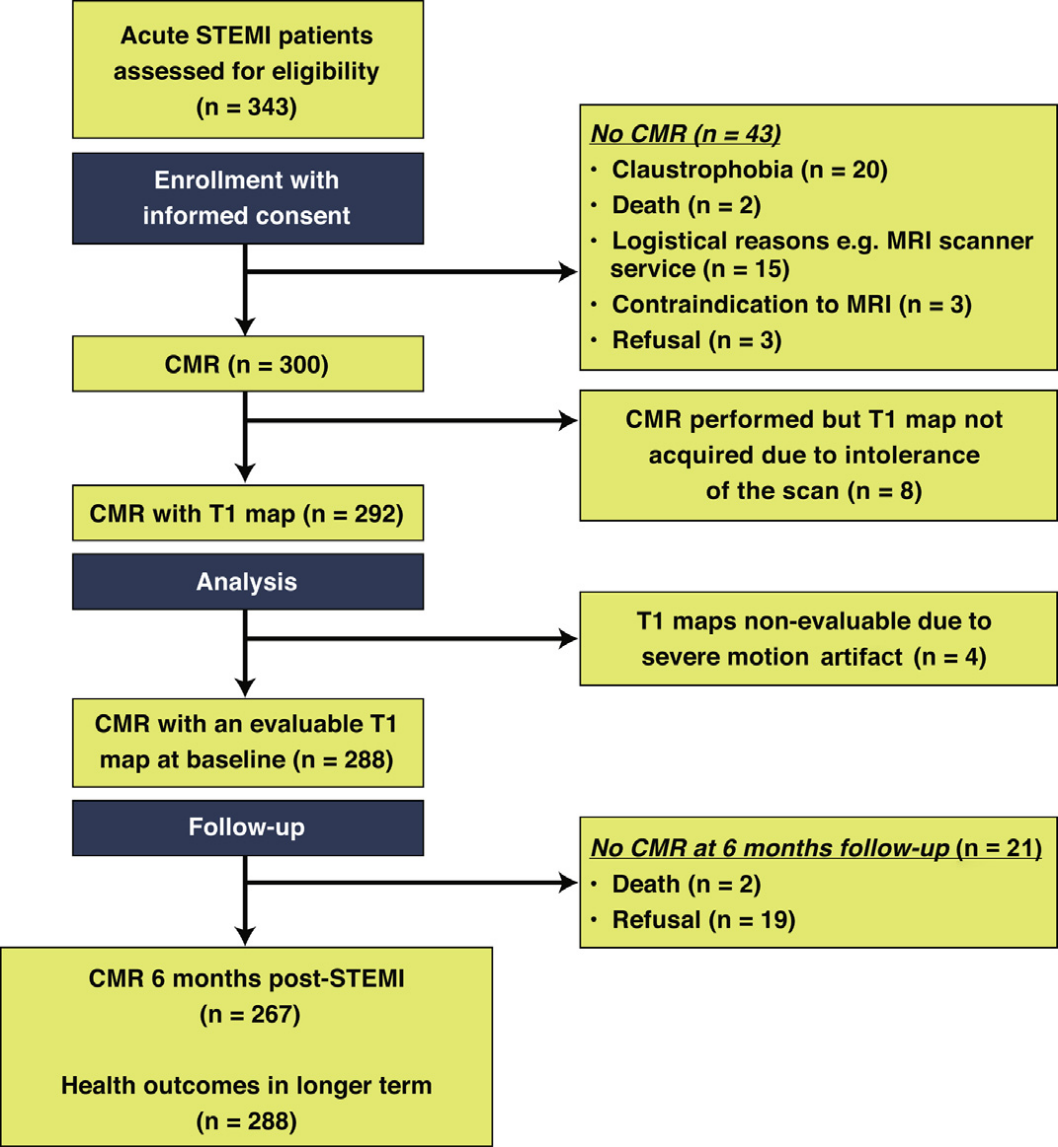
Flow Diagram of the Cohort Study CMR = cardiac magnetic resonance; other abbreviation as in Figure 1.

Of 343 STEMI patients referred for emergency PCI, 300 underwent serial CMR 2.2 ± 1.9 days and 6 months after hospital admission (Figure 2). A total of 292 STEMI patients had a CMR assessment of native T1 and 288 (99%) had evaluable T1 data (Figure 1, Figure 2). Each of these patients (n = 288) had vital status assessed at least 18 months after enrollment, and CMR follow-up at 6 months was achieved in 267 (91%) patients. The flow diagram for the study, including the reasons for nonattendance, is shown in Figure 2.

### Patient characteristics

The characteristics of the patients with evaluable native T1 CMR data (n = 288) are shown in Table 1. The characteristics of those patients with missing follow-up information are described in Online Table 1. The mean age was 59 ± 12 years, and 74% were male. Remote zone native T1 was inversely associated with male sex. A total of 158 patients (55%) had incomplete or no resolution of ST-segment elevation immediately following PCI. Circulating C-reactive protein (CRP) levels and leukocyte counts and their changes during serial testing in the first 2 days post-MI are described in Table 1 and Online Table 2, respectively. Angiotensin-converting enzyme inhibitors and beta-blockers were prescribed during the initial hospitalization in 285 (99%) and 278 (96.5%) of patients, respectively.

**Table 1:** Clinical and Angiographic Characteristics of 288 Patients With STEMI Who Had CMR With Evaluable Myocardial Native T1 Maps

	All Patients (N = 288)	STEMI Patient Group in Tertiles, Remote Zone Native T1 at Baseline			p Value∗
		≤951 ms (n = 96)	>951 to ≤969 ms (n = 96)	>969 ms (n = 96)	
Age, yrs	59 ± 12	59 ± 11	58 ± 12	60 ± 11	0.727
Male	211 (73)	78 (81)	73 (76)	60 (62)	0.011
BMI, kg/m^2^	29 ± 5	29 ± 4	29 ± 5	29 ± 5	0.910
Hypertension	93 (32)	26 (27)	33 (34)	34 (35)	0.418
Current smoking	177 (62)	55 (57)	61 (64)	61 (64)	0.640
Hypercholesterolemia	82 (28)	24 (25)	26 (27)	32 (33)	0.415
Diabetes mellitus†	32 (11)	12 (12)	13 (14)	7 (7)	0.354
Previous angina	34 (12)	11 (12)	10 (10)	13 (14)	0.850
Previous myocardial infarction	23 (8)	4 (4)	7 (7)	12 (12)	0.119
Previous PCI	16 (6)	4 (4)	6 (6)	6 (6)	0.852
Presenting characteristics					
Heart rate, beats/min	78 ± 17	77 ± 16	77 ± 17	80 ± 16	0.526
Systolic blood pressure, mm Hg	136 ± 24	134 ± 25	139 ± 25	134 ± 23	0.286
Diastolic blood pressure, mm Hg	79 ± 14	78 ± 12	82 ± 14	76 ± 15	0.062
Time from symptom onset to reperfusion, min	254 ± 217	254 ± 213	227 ± 212	283 ± 225	0.226
Ventricular fibrillation‡	20 (7)	7 (7)	7 (7)	7 (6)	1.000
Heart failure Killip class§					
I	205 (71)	73 (76)	73 (76)	59 (62)	
II	64 (22)	19 (20)	17 (17)	29 (30)	0.142
III or IV	19 (7)	4 (4)	7 (7)	8 (8)	
ECG					
ST-segment elevation resolution post-PCI					
Complete, ≥70%	129 (45)	54 (56)	42 (44)	33 (34)	
Incomplete, 30% to <70%	115 (40)	29 (30)	41 (43)	45 (47)	0.036
None, ≤30%	43 (15)	13 (14)	12 (13)	18 (19)	
Coronary angiography					
Reperfusion strategy					
Primary PCI	268 (93)	86 (90)	90 (94)	92 (96)	
Rescue PCI (failed thrombolysis)	13 (4)	6 (6)	4 (4)	3 (3)	0.527
Successful thrombolysis	7 (2)	4 (4)	2 (2)	1 (1)	
Number of diseased arteries‖					
1	156 (54)	53 (55)	57 (59)	46 (48)	
2	84 (29)	30 (31)	26 (27)	28 (29)	0.388
3	42 (15)	10 (10)	12 (12)	20 (21)	
Left main	6 (2)	3 (3)	1 (1)	2 (2)	
Culprit artery					
Left anterior descending	108 (38)	32 (33)	35 (36)	41 (43)	
Left circumflex	51 (18)	11 (12)	22 (23)	18 (19)	0.081
Right coronary	129 (45)	53 (55)	39 (41)	37 (38)	
TIMI coronary flow grade pre-PCI					
0/1	208 (72)	62 (65)	73 (76)	73 (76)	
2	52 (18)	20 (21)	14 (15)	18 (19)	0.158
3	28 (10)	14 (15)	9 (9)	5 (5)	
TIMI coronary flow grade post-PCI					
0/1	3 (1)	1 (1)	1 (1)	1 (1)	
2	13 (4)	4 (4)	5 (5)	4 (4)	1.000
3	272 (94)	91 (95)	90 (94)	91 (95)	
Initial blood results on admission¶					
C-reactive protein, mg/l Range	3.0 (2.0–7.0) 0.0–265.0	3.0 (2.0–5.0) 0.0–43.0	4.0 (2.0–7.0) 1.0–125.0	5.0 (3.0–10.8) 1.0–265.0	0.034
Leukocyte cell count, × 10^9^/l	12.4 (3.5)	11.5 (2.9)	13.2 (3.7)	12.4 (3.6)	0.002
Neutrophil count, × 10^9^/l	9.6 (3.2)	8.7 (2.8)	10.3 (3.4)	9.7 (3.3)	0.002
Monocytes, × 10^9^/l	0.8 (0.3)	0.9 (0.4)	0.8 (0.4)	0.9 (0.4)	0.382

Values are mean ± SD, n (%), median (IQR), or range. The patients are grouped according to tertiles of remote zone native T1 (ms) at baseline. p values were obtained from 1-way analysis of variance, Kruskal-Wallis test, or Fisher test.

BMI = body mass index; CMR = cardiac magnetic resonance; ECG = electrocardiogram; IQR = interquartile range; PCI = percutaneous coronary intervention; STEMI = ST-segment elevation myocardial infarction; TIMI = Thrombolysis In Myocardial Infarction.

∗ The p value is for the association between clinical characteristic and tertiles of remote zone native T1 at baseline.

† Diabetes mellitus was defined as a history of diet-controlled or treated diabetes.

‡ Successfully electrically cardioverted ventricular fibrillation at presentation or during emergency PCI procedure.

§ Killip classification of heart failure after acute myocardial infarction: class I = no heart failure; class II = pulmonary rales or crepitations, a third heart sound, and elevated jugular venous pressure; class III = acute pulmonary edema; and class IV = cardiogenic shock.

‖ Multivessel coronary artery disease was defined according to the number of stenoses of at least 50% of the reference vessel diameter by visual assessment and whether or not there was left main stem involvement.

¶ The blood results on admission and their changes during the first 2 days after admission are described in Online Table 1. C-reactive protein levels were available for 281 participants.

### CMR findings

#### Initial CMR findings during the index hospitalization

Remote zone native T1 for patients grouped by tertiles was associated with male sex (p = 0.011), ST-segment resolution (p = 0.036), CRP level (p = 0.034), leukocyte count (p = 0.002), and neutrophil count (p = 0.002) (Table 1). Remote zone native T1 was not associated with the number of coronary arteries affected by a stenosis of ≥50% severity or the culprit artery type.

The CMR findings are summarized in Table 2, and case examples are shown in Figure 1. At baseline, the mean myocardial infarct size was 18 ± 13% of LV mass, and 51% of patients had late microvascular obstruction. Native T1 in the remote myocardium was lower than native T1 in the infarct zone (961 ± 25 ms vs. 1,097 ± 52 ms; p < 0.01), reflecting infarct zone edema.

**Table 2:** Comparison of CMR Findings at Baseline (n = 288) and at 6 Months (n = 267) in Patients With STEMI Grouped According to Tertiles of Remote Zone Native T1 Values (ms) at Baseline

	All Patients	STEMI Patient Group in Tertiles, Remote Zone Native T1 at Baseline			p Value
		≤951 ms (n = 96)	>951 to ≤969 ms (n = 96)	>969 ms (n = 96)	
CMR findings 2 days post-MI (n = 288)					
LV ejection fraction, %	55 ± 10	56 ± 10	56 ± 9	53 ± 10	0.041
LV end-diastolic volume, ml					
Men	162 ± 33	158 ± 29	158 ± 35	172 ± 34	0.017
Women	124 ± 25	117 ± 25	131 ± 25	124 ± 25	0.233
LV end-systolic volume, ml					
Men	76 ± 26	70 ± 23	72 ± 26	87 ± 27	<0.001
Women	55 ± 18	52 ± 19	59 ± 17	53 ± 17	0.476
LV mass, g					
Men	144 ± 30	140 ± 28	142 ± 28	152 ± 33	0.054
Women	99 ± 24	93 ± 25	108 ± 25	96 ± 22	0.064
Edema and infarct characteristics					
Area at risk, % of LV mass	32 ± 12	29 ± 12	32 ± 12	35 ± 11	0.045
Infarct size, % of LV mass	18 ± 13	16 ± 13	17 ± 13	20 ± 14	0.044
Myocardial salvage, % of LV mass	19 ± 9	18 ± 9	20 ± 9	18 ± 8	0.370
Myocardial salvage index, % of LV mass	63 ± 24	68 ± 26	64 ± 23	57 ± 23	0.013
Late microvascular obstruction present	145 (50)	41 (43)	51 (53)	53 (55)	0.192
Late microvascular obstruction, % of LV mass	2.7 ± 4.6	1.6 ± 3.3	3.5 ± 5.4	3.2 ± 4.8	0.021
Myocardial native T1 values					
T1 remote myocardium (all participants), ms	961 ± 25	935 ± 12	960 ± 6	988 ± 17	<0.001
Men, ms	959 ± 25	935 ± 12	960 ± 6	988 ± 17	<0.001
Women, ms	968 ± 25	938 ± 11	961 ± 6	989 ± 18	<0.001
T1 infarct zone, ms	1,097 ± 52	1,077 ± 44	1,100 ± 52	1,115 ± 52	<0.001
T1 hypointense core present	160 (56)	50 (52)	52 (54)	58 (60)	0.483
T1 hypointense infarct core, ms	997 ± 57	986 ± 51	1,001 ± 64	1,003 ± 56	0.269
CMR findings 6 months post-MI (n = 267)					
LV ejection fraction at 6 months, %	62 ± 9	64 ± 8	63 ± 8	60 ± 11	0.010
LV end-diastolic volume at 6 months, ml					
Men	168 ± 37	160 ± 32	163 ± 32	182 ± 43	0.007
Women	128 ± 29	125 ± 20	128 ± 30	128 ± 33	0.995
LV end-systolic volume at 6 months, ml					
Men	66 ± 30	58 ± 21	63 ± 24	82 ± 39	<0.001
Women	46 ± 17	46 ± 16	48 ± 19	45 ± 18	0.895

Values are mean ± SD or n (%). Area at risk was measured with T2 mapping. p values were obtained from 1-way analysis of variance, Kruskal-Wallis test, or Fisher test. Three T1 maps (basal-, mid-, and distal-ventricular levels) were measured in each patient (n = 876 T1-maps overall), and 93% of these maps were suitable for analysis. Overall, 20 patients (6.8%) had poor-quality T1 maps and 4 patients (1.3%) had no evaluable T1 maps (Figure 1). Forty-two T1 maps were unsuitable for analysis because of steady-state free precession off-resonance artifacts, and 19 of these T1 maps were also affected by motion artifacts. Remote zone native T1 values were higher than T1 values in infarct tissue (p < 0.001) and in infarct core (p < 0.001).

LV = left ventricular; MI = myocardial infarction; other abbreviations as in Table 1.

#### CMR findings at 6 months

The CMR findings at 6 months are described in Table 2.

#### Myocardial native T1 in STEMI patients and healthy volunteers

Fifty healthy volunteers (52% male; mean age 54 ± 13 years) also underwent CMR. At the midventricular level, mean remote zone native T1 was similar in STEMI patients (961 ± 25 ms) and healthy volunteers (958 ± 24; p = 0.314) (Online Appendix). When described in tertiles (≤951, >951 to ≤969, and >969 ms), the upper tertile of remote zone native T1 had values that overlapped with T1 values observed in the infarct zone (Table 2).

The results of intraobserver and interobserver agreement of T1 measurements are shown in Online Figure 1.

### LV outcomes at 6 months post-MI

At 6 months, LV end-diastolic volume increased on average by 5 ± 25 ml (Table 2). Adverse remodeling, defined as an increase in LV end-diastolic volume by ≥20%, occurred in 30 patients (12%).

### Baseline associates of myocardial remote zone native T1

The univariable and multivariable baseline associates of remote zone native T1 are described in Online Table 3 and Table 3, respectively. In stepwise linear regression using AIC, incomplete ST-segment resolution (regression coefficient 9.42; 95% confidence interval [CI]: 2.37 to 16.47; p = 0.009) and a 1-U increase in log of the initial CRP concentration (3.01; 95% CI: 0.016 to 5.55; p = 0.038) were independently associated with T1. Myocardial remote zone native T1 was approximately 10 ms higher on average in patients with ECG evidence of reperfusion injury. Native T1 in the remote zone increased by approximately 10 ms, on average, for every 1 × 10^9^/l increase in peak monocyte count within 2 days of admission (10.20; 95% CI: 0.74 to 19.67; p = 0.035).

**Table 3:** Association of Patient Characteristics With Native T1 (ms) in Remote Myocardium in Univariable and Multivariable Stepwise Regression Analyses (n = 288)

Multiple Stepwise Regression	Coefficient (95% CI)	p Value
Including patient characteristics and angiographic data		
Male	−9.93 (−16.23 to −3.62)	0.002
Previous MI	11.38 (1.10 to 21.67)	0.030
Killip class IV	28.64 (1.00 to 56.28)	0.042
No ST-segment resolution	12.05 (3.67 to 20.43)	0.005
Incomplete ST-segment resolution	9.30 (3.20 to 15.39)	0.003
Including patient characteristics, angiographic data, and CRP		
Male	−9.54 (−16.62 to −2.47)	0.008
No ST-segment resolution	10.54 (1.11 to 19.97)	0.029
Incomplete ST-segment resolution	9.46 (2.41 to 16.50)	0.009
Log initial CRP	2.75 (−0.09 to 5.58)	0.057
Including patient characteristics, angiographic data, initial CRP, and LV end-diastolic volume		
Male	−12.74 (−20.69 to −4.79)	0.002
BMI, kg/m^2^	−0.63 (−1.33 to 0.08)	0.083
No ST-segment resolution	10.12 (0.69 to 19.56)	0.036
Incomplete ST-segment resolution	9.42 (2.37 to 16.47)	0.009
Log initial CRP	3.01 (0.016 to 5.85)	0.038
LV end-diastolic volume	0.10 (−0.01 to 0.21)	0.084
Including patient characteristics, angiographic data, peak monocyte count, and LV end-diastolic volume		
Male	−14.31 (−22.33 to −6.29)	<0.001
Previous myocardial infarction	−0.63 (−1.33 to 0.08)	0.083
No ST-segment resolution	10.09 (0.72 to 19.47)	0.035
Incomplete ST-segment resolution	9.23 (2.29 to 16.17)	0.009
Peak monocyte count, × 10^9^/l	10.20 (0.74 to 19.67)	0.035
LV end-diastolic volume	0.10 (−0.01 to 0.21)	0.088

The coefficient (95% CI) indicates the magnitude and direction of the difference in remote zone T1 (ms) for the patient characteristic (binary or continuous). For example, on average, remote zone native T1 (ms) 2 days post-MI was −9.93 (95% CI: −16.23 to −3.62) lower for male versus female, and native T1 (ms) was 0.14 (95% CI: 0.01 to 0.26) higher for each 1-ml increase in LV end-systolic volume. Previous MI affecting a territory of remote myocardium could alter native T1, which could be a confounding factor in the multivariable analysis of associates of remote zone native T1. In a sensitivity analysis involving backward stepwise variable selection, removing previous MI had no effect on the results of the multivariable models.

CI = confidence interval; CRP = C-reactive protein; other abbreviations as in Table 1, Table 2.

### Myocardial tissue characteristics as a marker of subsequent LV remodeling

Myocardial remote zone native T1 measured 2 days post-MI was multivariably associated with LV end-diastolic volume at 6 months (Table 4), including in patients without microvascular obstruction (n = 121) (Online Table 4). Remote T1 was also multivariably associated with change in LV end-diastolic volume from baseline (n = 267) (Online Figure 2, Online Table 5) after adjustment for initial LV end-diastolic volume, infarct size, and microvascular obstruction.

**Table 4:** Multivariable Association of Patient Characteristics and Angiographic and CMR Findings With LV End-Diastolic Volume at 6 Months Post-STEMI (n = 267)∗

	Coefficient (95% CI)	p Value
Remote zone native T1, ms	0.16 (0.05 to 0.27)	0.005
LV end-diastolic volume at baseline, ml	0.69 (0.59 to 0.79)	<0.001
Infarct size, % LV mass	0.83 (0.56 to 1.11)	<0.001
Male	17.82 (10.78 to 24.86)	<0.001
BMI, kg/m^2^	0.67 (0.02 to 1.33)	0.045
Hypercholesterolemia	−6.85 (−13.65 to −0.05)	0.048
Sustained ventricular arrhythmia	12.36 (1.33 to 23.39)	0.028
Reperfusion mode: successful thrombolysis	48.91 (5.71 to 92.10)	0.027

The coefficient and 95% CI indicate the magnitude and direction of the difference in LV end-diastolic volume (ml) at follow-up for the patient characteristic (binary or continuous). For example, on average, LV end-diastolic volume (ml) at follow-up was 0.16 (95% CI: 0.05 to 0.27) higher for each 1-ms increase in remote zone native T1 measured by CMR at baseline. The univariable associations with LV end-diastolic volume at 6 months are described in the Online Appendix.

Abbreviations as in Table 1, Table 2, Table 3.

∗ When area at risk was included instead of infarct size, remote zone T1 was also associated with LV end-diastolic volume at follow-up (0.25; 95% CI: 0.09 to 0.41; p = 0.002).

### Remote zone native T1 early post-MI and N-terminal pro–B-type natriuretic peptide, a biochemical measure of LV remodeling, at 6 months

Blood samples had been collected in STEMI patients enrolled during office hours, and N-terminal pro–B-type natriuretic peptide (NT-proBNP) results were available in 151 patients. The clinical and CMR characteristics of these patients were similar to those of the whole cohort (Online Tables 6 and 7).

**Figure 3:**
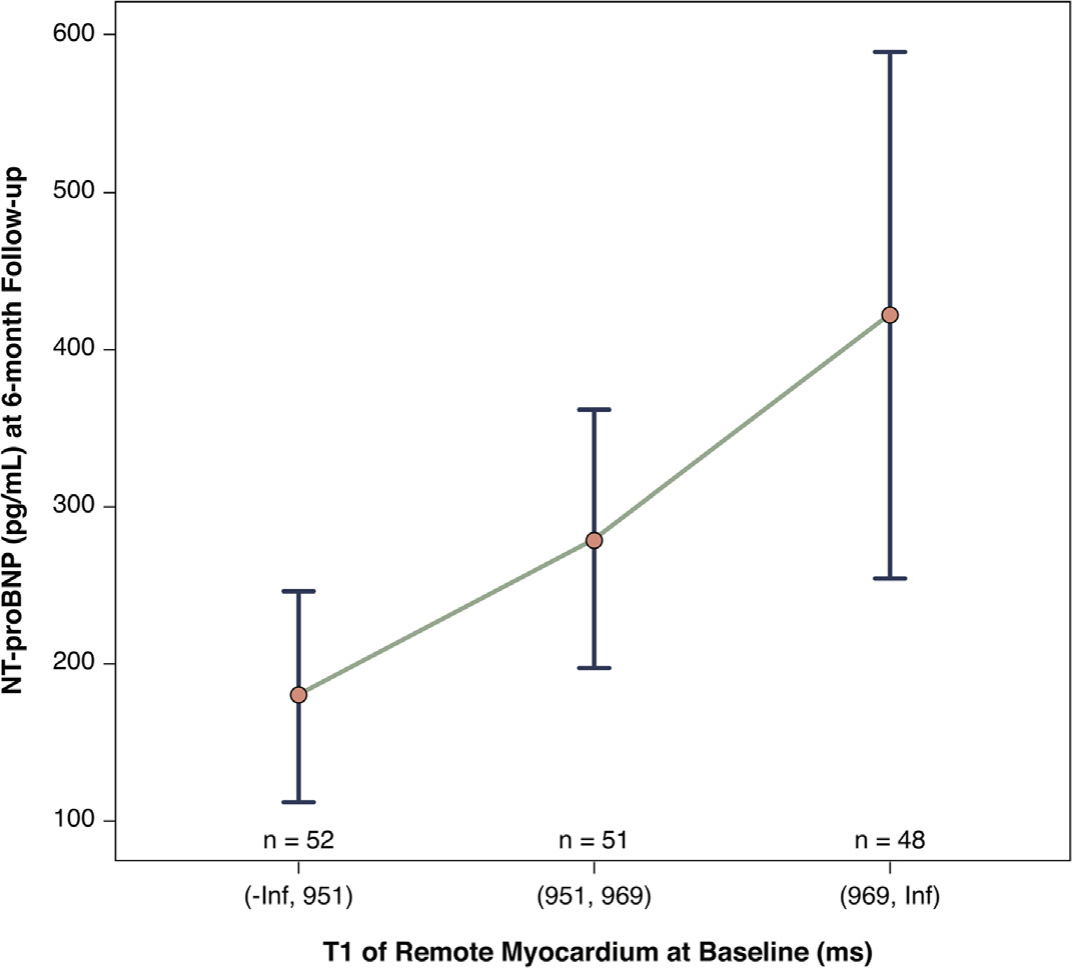
Remote Zone Native T1 and NT-proBNP 6 Months Post-MI Remote zone native T1 (ms) at baseline was associated with N-terminal pro–B-type natriuretic peptide (NT-proBNP) (median [interquartile range]) after 6 months (n = 151 patients with ST-segment elevation myocardial infarction) (Online Appendix). MI = myocardial infarction.

Remote zone native T1 and NT-proBNP levels were not associated at baseline. By contrast, the concentrations of NT-proBNP at 6 months differed between the lowest versus highest tertiles of remote zone native T1 at baseline (Figure 3). This relationship remained after adjustment for LV ejection fraction and volumes. Specifically, remote zone native T1 at baseline in tertiles (lowest tertile = reference; intermediate tertile p = 0.145; upper tertile p = 0.013) was associated with log NT-proBNP at 6 months after adjustment for NT-proBNP at baseline (0.01 on log scale; 95% CI: 0.00 to 0.02; p = 0.049), independently of LV ejection fraction (p = 0.064) and LV end-diastolic volume at baseline (p = 0.046) (Figure 3).

### Myocardial remote zone T1 and health outcomes in the longer term

Follow-up data were obtained in 100% of the 288 patients. The median duration of follow-up was 845 days (minimum to maximum post-discharge censor duration: 598 to 1,098 days). Thirty-nine patients (13.5%) experienced MACE. These events included 3 cardiac deaths, 14 hospitalizations for recurrent MI, and 22 episodes of heart failure (Killip class 3 or 4 heart failure or defibrillator implantation). Of 39 patients with MACE, 20 (6.9%) experienced MACE post-discharge. Myocardial remote zone native T1 was associated with all MACE (p = 0.008). Remote zone native T1 was associated with MACE post-discharge (1-ms increase in native T1: hazard ratio: 1.016; 95% CI: 1.000 to 1.032; p = 0.048), including after adjustment for LV end-diastolic volume at baseline (p = 0.051) or the temporal changes in CRP concentration (p = 0.041) and circulating numbers of monocytes (p = 0.036) and other leukocyte subpopulations (all p < 0.05) (Online Table 8).

Remote zone native T1 was also associated with all-cause death or heart failure hospitalization (n = 30 events during admission and post-discharge; 1.014; 95% CI: 1.000 to 1.028; p = 0.049), including after adjustment for LV end-diastolic volume (p = 0.055) and change in LV ejection fraction (p = 0.028). In an exploratory analysis instigated during peer review, a native T1 cutoff of 969 ms had a negative predictive value of 0.84 for all cardiovascular outcomes combined (Online Appendix).

The net reclassification index and C-index indicated that adding remote zone native T1 did not alter the predictive value of the models (Online Appendix).

## Discussion

We used CMR to measure native T1 in myocardial regions of interest in a large cohort of STEMI survivors enrolled acutely during usual care, and we assessed the pathophysiology and prognostic importance of remote zone tissue.

The main findings of our study were:

1. The upper tertile of remote zone native T1 values approximated infarct zone T1 values.
2. Remote myocardial native T1 measured in STEMI survivors on average 2 days post-MI was independently associated with systemic inflammation and the size of MI. The temporal changes in circulating monocyte cell numbers early post-MI were independently associated with remote zone native T1 and subsequent LV remodeling at 6 months, supporting a potential mechanistic link between inflammation and adverse LV remodeling.
3. Remote myocardial native T1 was associated with changes in LV end-diastolic volume 6 months post-MI, independent of the initial severity of MI, as revealed by microvascular obstruction and LV end-diastolic volume 2 days post-MI. Remote zone native T1 was also associated with NT-proBNP concentrations at 6 months.
4. Remote myocardial native T1 was independently associated with post-discharge adverse cardiac events, including MACE and all-cause death and heart failure hospitalization during longer-term follow-up.
5. A native T1 value <969 ms (i.e., below the upper tertile) had a high negative predictive value for adverse cardiovascular outcomes.

The potential clinical utility of a cutoff for the middle versus upper tertile of remote zone T1 for risk assessment merits further prospective assessment.

Quantitative T1 mapping with CMR has provided regional measurements of tissue characteristics that were temporally associated with reperfusion injury (persistent ST-segment elevation on the ECG), the size of MI (area at risk and infarct size), inflammation, and LV and clinical outcomes in the longer term. Native T1 is strongly determined by tissue water content and cellularity 14, 15, and myocardial native T1 increases with inflammatory cell infiltration, as revealed by histopathology in patients who underwent cardiac transplant with features of acute rejection (37). An increase in myocardial remote zone native T1 early post-MI most likely reflects edema and hypercellularity 9, 10, 11, 37. In fact, the upper tertile of remote zone native T1 overlapped with native T1 values in the infarct zone (Table 2) and native T1 values in myocardial edema, as reported by Ferreira et al. (38). An alternative explanation could be that remote zone ischemia could cause native T1 elevation through microvascular dilation and increased blood volume (39); however, there was no relationship between the number of diseased coronary arteries and remote zone native T1 (Table 1). Remote zone native T1 and NT-proBNP levels at baseline were not associated, implying that LV wall stress alone is unlikely to be a determinant of native T1 acutely, which is in contrast to the striking associations that we have observed between remote zone native T1 and reperfusion injury and inflammation. Reperfusion injury was relatively common in our cohort (Table 1), perhaps reflecting an unselected real-life STEMI population.

Our clinical findings are consistent with pre-clinical observations that monocytes participate in remote zone inflammation and remodeling 5, 6, 7, 8, 9, 10 (Online Appendix). Remote zone native T1 was independently associated with LV remodeling at 6 months post-STEMI as revealed by CMR and NT-proBNP levels. These observations explain why native T1 in remote myocardium early post-MI might influence longer-term prognosis because adverse remodeling is strongly associated with reduced survival post-MI (2). Acute inflammation early post-STEMI may be a target for therapy 5, 13. Future studies should assess whether or not remote zone T1 might be influenced by therapeutic interventions in STEMI patients and, therefore, potentially may represent a quantitative surrogate biomarker of therapeutic effect in the heart.

Because a gadolinium contrast agent is not needed for native T1 CMR, this biomarker may be informative in a broader range of patients with MI, including those with severe renal dysfunction for whom contrast CMR is contraindicated. This hypothesis merits further prospective assessment in future studies. Our study does not permit inference on causality, and other interpretations of our data are possible. A higher T1 value of remote myocardium may indicate patients with more diffuse fibrosis, possibly reflecting underlying microvascular disease and an increased susceptibility to inflammation injury. Although we did not systematically assess for remote zone ischemia using fractional flow reserve or stress CMR, we think that the absence of an association with multivessel coronary disease counts against ischemia as the main cause of native T1 elevation in the myocardial remote zone.

### Study limitations

We do not have information on wall stress, which could influence remote zone pathophysiology. Future research should determine the natural history and clinical significance of native T1 changes during longer-term follow-up.

## Conclusions

In acute STEMI survivors, remote zone native T1 was temporally linked with reperfusion injury and inflammation and independently associated with LV remodeling and adverse cardiovascular outcomes. The upper T1 tertile represents a potential cutoff for prognostication that merits further study.

Perspectives **COMPETENCY IN MEDICAL KNOWLEDGE:** In acute STEMI survivors, CMR of remote myocardial tissue changes, as revealed by measurement of native T1, provides information that is linked with reperfusion injury and inflammation. Remote zone native T1 is independently associated with LV remodeling and adverse cardiovascular outcomes.

**TRANSLATIONAL OUTLOOK:** Elevated remote zone native T1 in the upper tertile may represent an imaging biomarker for prognostication in survivors of acute STEMI, including for adverse LV remodeling and all-cause death and heart failure in the longer term. This possibility merits further prospective validation to confirm or refute the potential utility of this imaging biomarker in clinical practice.
