# Supplementary material for: Pathophysiology of LV Remodeling in Survivors of STEMI: Inflammation, Remote Myocardium, and Prognosis
Source: JACC Cardiovasc Imaging. 2015 Jul;8(7):779–89. doi: 10.1016/j.jcmg.2015.03.007 (PMC4509710; doi:10.1016/j.jcmg.2015.03.007)
Supplement: Online Data [file mmc1.docx]

# Supplementary Methods

**Pathophysiology of left ventricular remodeling in survivors of ST-elevation myocardial infarction: inflammation, remote myocardium and prognosis.**

# ClinicalTrials.gov registration NCT02072850Table of contents

[Setting and study populations 3](#_Toc413351139)

[Coronary angiogram acquisition and analyses 4](#_Toc413351140)

[Percutaneous coronary intervention 4](#_Toc413351141)

[Angiographic analysis 5](#_Toc413351142)

[Outcome definitions 5](#_Toc413351143)

[CMR acquisition and analyses 6](#_Toc413351144)

[Electrocardiogram 9](#_Toc413351145)

[Biochemical and hematologic measurement of inflammation 10](#_Toc413351146)

[Hematologic measurement of inflammation 11](#_Toc413351147)

[Statistics 12](#_Toc413351148)

[Trial Management 14](#_Toc413351149)

[Health outcomes and their definitions 14](#_Toc413351150)

[References 15](#_Toc413351151)

[Clinical Event Adjudication Charter 16](#_Toc413351152)

[Objective of the Event Adjudication Charter 18](#_Toc413351156)

[Adverse Event definitions 20](#_Toc413351158)

[Clinical data to be provided 44](#_Toc413351159)

# Setting and study populations

### STEMI patients

Screening, enrolment, and data collection were prospectively performed by cardiologists in the cardiac catheterization laboratories of the Golden Jubilee National Hospital, Glasgow, United Kingdom. This hospital is a regional referral center for primary and rescue percutaneous coronary intervention (PCI). The hospital provides clinical services for a population of 2.2 million. A screening log was recorded, including patients who did not participate in the cohort study.

### Healthy volunteers

The purpose of including healthy volunteers was to collect normative reference data for myocardial native T1 in individuals without prior cardiovascular disease or therapy and who were reasonably representative of the population of individuals from whom the STEMI patients were drawn. Second, the reference native T1 values were required to be measured on the same CMR scanner and with the same protocol that was used for the STEMI patients including during the same time-period.

Healthy volunteers were invited to participate by placing adverts in public buildings (e.g. hospital, University) and through personal contacts of the researchers. Matching and selection of the healthy volunteers was done by the researchers in order to reflect the age and gender distribution of the STEMI patients. The healthy volunteers were resident in the same catchment area as the STEMI population. Fifty age- and gender-matched healthy volunteers who had a normal ECG and no prior history of cardiovascular disease or therapy underwent CMR during the same time period. The absence of late gadolinium enhancement (myocardial fibrosis or scar) was determined qualitatively by visual assessment, and the absence of late gadolinium enhancement was a requirement for inclusion of the volunteer in this analysis.

The rationale for including healthy volunteers in this study is as follows. First, native T1 values may vary between CMR scanners and so a local reference range for native T1 is recommended in CMR guidelines [32]. Second, native T1 may vary spatially in the heart and therefore, since the focus of our study was to assess native T1 in myocardium remote from the infarct zone, we aimed to collect native T1 values in different segments of the heart in order to compare the remote zone native T1 values from STEMI patients with reference spatially matched remote zone native T1 values in age- and gender-matched healthy volunteers. Myocardial native T1 values were regionally segmented in regions-of-interest and summarized according to the AHA model [38].

# Coronary angiogram acquisition and analyses

Coronary angiograms were acquired during usual care with cardiac catheter laboratory X-ray (Innova®) and IT equipment (Centricity®) made by GE Healthcare. The coronary anatomy and disease characteristics of study participants were described based on the clinical reports of the attending cardiologist.

# Percutaneous coronary intervention

Consecutive admissions with acute ST-elevation myocardial infarction (STEM) referred for emergency percutaneous coronary intervention (PCI) were screened for the inclusion and exclusion criteria. During ambulance transfer to the hospital, the patients received 300 mg of aspirin, 600 mg of clopidogrel and 5000 IU of unfractionated heparin [29,30]. The initial primary PCI procedure was performed using radial artery access. A conventional approach to primary PCI was adopted in line with usual care in our hospital [29,30]. Conventional bare metal and drug eluting stents were used in line with guideline recommendations and clinical judgement. The standard transcatheter approach for reperfusion involves minimal intervention with aspiration thrombectomy only or minimal balloon angioplasty (e.g. a compliant balloon sized according to the reference vessel diameter and inflated at 4-6 atmospheres 1-2 times). During PCI, glycoprotein IIbIIIa inhibitor therapy was initiated with high dose tirofiban (25 μg/kg/bolus) followed by an intravenous infusion of 0.15 μg/kg/min for 12 hours, according to clinical judgement and indications for bail-out therapy [30]. No reflow was treated according to contemporary standards of care with intra-coronary nitrate (i.e. 200 μg) and adenosine (i.e. 30 – 60 μg) [30], as clinically appropriate. In patients with multivessel coronary disease, multivessel PCI was not recommended, in line with clinical guidelines [30]. The subsequent management of these patients was symptom-guided.

# Angiographic analysis

The coronary anatomy and disease characteristics of study participants were described based on the clinical reports of the attending cardiologist.

# Outcome definitions

Coronary blood flow can be described based on the visual assessment of coronary blood flow revealed by contrast injection into the coronary arteries [30].

| TIMI Coronary Flow Grade |  |
| --- | --- |
| 0 | No flow |
| 1 | Minimal flow past obstruction |
| 2 | Slow (but complete) filling and slow clearance |
| 3 | Normal flow and clearance |

# CMR acquisition and analyses

## CMR acquisition

CMR was performed on a Siemens MAGNETOM Avanto (Erlangen, Germany) 1.5-Tesla scanner with a 12-element phased array cardiac surface coil. T1 maps were acquired in 3 short-axial slices (basal, mid and apical), using an optimized modified look-locker inversion-recovery (MOLLI) investigational prototype sequence [24,25] before contrast administration (Supplementary Methods). The MOLLI T1 cardiac-gated acquisition involved three inversion-recovery prepared look locker experiments combined within one protocol (3 (3) 3 (3) 5) [25]. The CMR parameters were: bandwidth ~1090 Hz/pixel; flip angle 35°; echo time (TE) 1.1 ms; T1 of first experiment 100 ms; TI increment 80 ms; matrix 192 x 124 pixels; spatial resolution 2.2 x 1.8 x 8.0 mm; slice thickness 8 mm; scan time 17 heartbeats .

T2 maps were acquired in contiguous short axis slices covering the whole ventricle, using an investigational prototype T2-prepared (T2P) TrueFisp sequence [34,35] (Supplementary Methods). Typical imaging parameters were: bandwidth ~947 Hz/pixel; flip angle 70°; T2 preparations: 0 ms, 24 ms, and 55 ms respectively; matrix 160 x 105 pixels; spatial resolution 2.6 x 2.1 x 8.0 mm; slice thickness 8 mm.

Late gadolinium enhancement images covering the entire LV were acquired 10-15 minutes after intravenous injection of 0.15 mmol/kg of gadoterate meglumine (Gd^2+^-DOTA, Dotarem, Guebert S.A.) using segmented phase-sensitive inversion recovery (PSIR) turbo fast low-angle shot [36]. Microvascular obstruction was defined as a dark zone on early delayed enhancement imaging 1, 3, 5 and 7 minutes post-contrast injection and within an area of late gadolinium enhancement. Typical imaging parameters were: matrix = 192 x 256, flip angle = 25°, TE = 3.36 ms, bandwidth = 130 Hz/pixel, echo spacing = 8.7ms and trigger pulse = 2. The voxel size was 1.8 x 1.3 x 8 mm^3^. Inversion times were individually adjusted to optimize nulling of apparently normal myocardium (typical values, 200 to 300 ms).

## MR image analyses

The images were analyzed on a Siemens work-station by observers with at least 3 years CMR experience (N.A., D.C., I.M, S.R.). All of the images were reviewed by experienced CMR cardiologists (C.B., N.T.). LV dimensions, volumes and ejection fraction were quantified using computer assisted planimetry (syngo MR®, Siemens Healthcare, Erlangen, Germany). All scan acquisitions were spatially co-registered.

### T1 - standardized measurements in myocardial regions of interest

LV contours were delineated with computer assisted planimetry on the raw T1 image and copied onto the color-encoded spatially co-registered maps. Apical segments were not included because of partial volume effects. Particular care was taken to delineate regions of interest with adequate margins of separation from tissue interfaces prone to partial volume averaging such as between myocardium and blood [31,32,37]. Each T1 map image was assessed for the presence of artifacts relating to susceptibility effects, or cardio-respiratory motion. Each motion-corrected series was evaluated for image alignment. Each map was evaluated against the original images. When artifacts occurred the affected segments were not included in the analysis.

In STEMI patients, myocardial T1 values were segmented spatially and regions-of-interest were defined as (1) remote myocardium, (2) injured myocardium and (3) infarct core. The regions-of-interest were planimetered to include the entire area of interest with distinct margins of separation from tissue interfaces to exclude partial volume averaging. The remote myocardial region-of-interest was defined as myocardium 180º from the affected zone with no visible evidence of infarction, edema or wall motion abnormalities by inspecting corresponding contrast enhanced T1-weighted, T2-weighted and cine images, respectively. The infarct zone region-of-interest was defined as myocardium with pixel values (T1 or T2) >2 SD from remote myocardium on T2-weighted CMR [33,34]. The infarct core was defined as an area in the center of the infarct territory having a mean T1 value of at least 2 standard deviations below the T1 value of the periphery of the area-at-risk. A parameter was also created for native T1 in the infarct zone indexed to the remote zone.

In healthy volunteers, the mid-ventricular T1 map was segmented into 6 equal segments, using the anterior right ventricular-LV insertion point as the reference point [38]. T1 was measured in each of these segments, and regions-of-interest were planimetered distinct and separate from blood-pool and tissue interfaces. These segmental values were also averaged to provide one value per subject. Results are presented as average values for segments and slices.

### Infarct definition and size

The presence of acute infarction was established based on abnormalities in cine wall motion, rest first-pass myocardial perfusion, and delayed-enhancement imaging. In addition, supporting changes on the ECG and coronary angiogram were also required. Acute infarction was considered present only if late gadolinium enhancement was confirmed on both the axial and long axis acquisitions. The myocardial mass of late gadolinium (grams) was quantified using computer assisted planimetry and the territory of infarction was delineated using a signal intensity threshold of >5 standard deviations above a remote reference region and expressed as a percentage of total LV mass [31,37]. Infarct regions with evidence of microvascular obstruction were included within the infarct area and the area of microvascular obstruction was assessed separately and also expressed as a percentage of total LV mass.

### Area-at-risk

Area-at-risk was defined as LV myocardium with pixel values (T1/T2) >2 standard deviations from remote myocardium [4,27,28,39-41]. In order to assess the area-at-risk the epicardial and endocardial contours on the last corresponding T2-weighted raw image with an echo time of 55 ms were planimetered [34]. Contours were then copied to the map and corrected when necessary by consulting the SSFP cine images.

### Myocardial salvage

Myocardial salvage was calculated by subtraction of percent infarct size from percent area-at-risk [4,39-41]. The myocardial salvage index was calculated by dividing the myocardial salvage area by the initial area-at-risk.

### Adverse remodeling

Adverse remodeling was defined as an increase in LV end-diastolic volume ≥ 20% at 6 months from baseline [3].

### Reference ranges

Reference ranges used in the laboratory were 105 – 215 g for LV mass in men, 70 – 170 g for LV mass in women, 77 – 195 ml for LV end-diastolic volume in men, 52 – 141 ml for LV end-diastolic volume in women, 19 – 72 ml for LV end-systolic volume in men and 13 – 51 ml for LV end-systolic volume in women.

# Electrocardiogram

A 12 lead electrocardiogram (ECG) was obtained before coronary reperfusion and 60 minutes afterwards with Mac-Lab® technology (GE Healthcare) in the catheter laboratory and a MAC 5500 HD recorder (GE Healthcare) in the Coronary Care Unit. The ECGs were acquired by trained cardiology staff. The ECGs were de-identified and transferred to the local ECG management system. The ECGs were then analyzed by the University of Glasgow ECG Core Laboratory which is certified to ISO 9001: 2008 standards as a UKAS Accredited Organization.

The extent of ST-segment resolution on the ECG assessed 60 minutes after reperfusion compared to the baseline ECG before reperfusion [29] was expressed as complete (≥70%), incomplete (30% to < 70%) or none (≤30%).

# Biochemical and hematologic measurement of inflammation

Serial systemic blood sample were obtained immediately after reperfusion in the cardiac catheterization laboratory, and subsequently between 0600 - 0700 hrs each day during the initial in-patient stay in the Coronary Care Unit. C-reactive protein (CRP) was measured in an NHS hospital biochemistry laboratory using a particle enhanced immunoturbimetric assay method (Cobas C501, Roche),) and the manufacturers calibrators and quality control material, as a biochemical measure of inflammation. The high sensitive assay CRP measuring range is 0.1-250 mg/L. The expected CRP values in a healthy adult are < 5 mg/L, and the reference range in our hospital is 0 - 10 mg/L. A blood sample was routinely obtained in the cardiac catheter laboratory immediately following revascularization and then again at 0700 hrs on the first and second days after admission to hospital.

NT-proBNP, a biochemical measure of LV wall stress, was measured in a research laboratory using an electrochemiluminescence method (e411, Roche) and the manufacturers calibrators and quality control material. The limit of detection is 5 pg/ml. Long-term coefficient of variations of low and high controls are typically <5%, and were all within the manufacturers range.

# Hematologic measurement of inflammation

Leucocyte count and leucocyte sub-populations were measured as a hematologic measure of inflammation using sheath flow technology incorporating semi-conductor laser beam, forward and side scattered light (Sysmex XT200i and XT1800i for white blood cell and differential white blood cell counts, respectively). The linearity ranges for white blood cells was 0.00-440.0 x10(9) /L. The following are the normal ranges for full blood count parameters:

|  | **MALE** | **FEMALE** |
| --- | --- | --- |
| WBC x 10^9/L | 4.0 - 11.0 | 4.0 - 11.0 |
| RBC x 10^12/L | 4.50 - 6.50 | 3.80 - 5.80 |
| Hgb g/L | 130 - 180 | 115 - 165 |
| HCT L/L | 0.400 - 0.540 | 0.370 - 0.470 |
| MCV fL | 78 - 99 | 78 - 99 |
| MCH Pg | 27.0 - 32.0 | 27.0 - 32.0 |
| MCHC g/L | 310 - 360 | 310 - 360 |
| PLATELETS x 10^9/L | 150 - 400 | 150 - 400 |
| NEUTROPHILS x 10^9/L | 2.5 - 7.5 | 2.5 - 7.5 |
| LYMPHOCYTES x 10^9/L | 1.5 - 4.0 | 1.5 - 4.0 |
| MONOCYTES x 10^9/L | 0.2 - 0.8 | 0.2 - 0.8 |
| EOSINOPHILS x 10^9/L | 0.0 - 0.4 | 0.0 - 0.4 |
| BASOPHILS x 10^9/L | 0.01 - 0.10 | 0.01 - 0.10 |

A blood sample was routinely obtained in the cardiac catheter laboratory, immediately following revascularization and then again at 0700 on the first and second days after admission to hospital.

# Statistics

### Sample size calculation

The sample size of 300 was predetermined based on the incidence of infarct pathology (e.g. myocardial hemorrhage or microvascular obstruction) affecting at least one third of the cohort. With an estimated hemorrhage incidence of 33% at 48 h post-STEMI, 100 subjects would have evidence of myocardial hemorrhage and 200 subjects would not. The study would have 90% power at a 5% level of significance using a two sided two sample t-test to detect a between-group difference in mean LV end-systolic volume index of 4.65 ml/m2 equivalent to three eighths of a common standard deviation (or an effect size of 0.375). We predicted a between-group difference in mean LVESVI of 4.65 ml/m2 equivalent to three eighths of a common standard deviation (or an effect size of 0.375). We also estimated that at least 30 MACE events would occur based on a conservative estimate of the event rate (10-12%) at 18 months.

### Statistical analysis

Categorical variables were expressed as number and percentage of patients. Most continuous variables had a normal distribution and are therefore presented as means together with standard deviation. Differences between groups were assessed by the Student t test for continuous data with normal distribution, otherwise the nonparametric Wilcoxon rank sum test was used. Correlation analyses were Pearson or Spearman tests, as indicated. Random effects models were used to compute inter-rater reliability measures (intra-class correlation coefficient (ICC)) for the reliability of CMR parameters measured independently by 2 observers in 20 randomly selected patients from the whole cohort.

Univariable and multivariable regression analyses were performed to identify correlates of T1 values for (1) remote myocardium, (2) injured myocardium and (3) the infarct core in all patients and in patients without late microvascular obstruction [3]. Stepwise selection methods with Akaike information criteria (AIC) were used to determine the most important associations. The CMR parameters that were all highly correlated with one another were included in multiple stepwise regression models with patient characteristics, angiographic data and blood results separately in order to reduce multi-collinearity. Separate multivariable analyses were performed for (A) patient characteristics and angiographic data and (B) CMR data. CMR parameters, which were all highly correlated with one another, were included separately in multiple stepwise regression models with patient characteristics and angiographic data to reduce multicollinearity. Non-linear relationships between T1 values in regions of interest and LV ejection fraction and end-diastolic volume were assessed with cubic splines and Loess plots. Binary logistic regression models were used to identify predictors of adverse remodeling at 6-month follow-up. Kaplan-Meier and Cox proportional hazards methods were used to identify potential predictors of MACE and all-cause death/heart failure events.

The net reclassification index and C-statistic were determined for the effect of adding remote zone native T1 to the prognostic models for binary outcomes including adverse LV remodeling revealed by CMR at 6 months and health outcomes in the longer term. A p-value > 0.05 indicates the absence of a statistically significant effect.

# **Trial Management**

The study was conducted in line with Guidelines for Good Clinical Practice (GCP) in Clinical Trials. <http://www.mrc.ac.uk/documents/pdf/good-clinical-practice-in-clinical-trials/>

Trial management included a Trial Management Group, and an independent Clinical Trials Unit. Day to day study activity was coordinated by the Trial Management Group who was responsible to the Sponsor which was responsible for overall governance and that the trial was conducted according to GCP standards.

Clinical events were assessed and validated by an independent cardiologist (A.M.) who had access to relevant source clinical data. This cardiologist followed an agreed charter and he was blinded to all of the other clinical data.

# Health outcomes and their definitions

A comprehensive definition of adverse events [46,47] and their adjudication is detailed in the Clinical Event Committee Charter.

We pre-specified adverse health outcomes that are pathophysiologically linked with STEMI. The primary composite outcome was (1) major adverse cardiac events (MACE) defined as cardiac death, non-fatal myocardial infarction (MI) or hospitalization for heart failure (Supplementary Methods). All-cause death or heart failure hospitalization was a secondary outcome.

After enrolment, research staff screened for MACE by checking the national electronic medical records of the study participants and by contacting the patients and their primary and secondary care physicians, as appropriate. Each event was reviewed by a cardiologist who was independent of the research team and blinded to all of the clinical and CMR data. The adverse events were defined according to standard guidelines and categorized as having occurred during the index admission or post-discharge. All study participants were followed-up for a minimum of 18 months after discharge.

# References

46. Hicks KA, Hung HMJ, Mahaffey KW, *et al*; on behalf of the Standardized Data Collection for Cardiovascular Trials Initiative Standardized Definitions for End Point Events in Cardiovascular Trials. <http://www.cdisc.org/stuff/contentmgr/files/0/2356ae38ac190ab8ca4ae0b222392b37/misc/cdisc_november_16__2010.pdf>

47. Thygesen K, Alpert JS, Jaffe AS, Simoons ML, Chaitman BR, White HD; Joint ESC/ACCF/AHA/WHF Task Force for the Universal Definition of Myocardial Infarction. Third universal definition of myocardial infarction. Circulation. 2012; 126:2020-35.

# Clinical Event Adjudication Charter

# Detection and Significance of Heart Injury in ST Elevation Myocardial Infarction –

# The BHF MR-MI study

#

# NCT02072850

## Rationale for the independent adjudication of clinical events

As a measure of enhanced Pharmacovigilance (PV) and Good Clinical Practice, a cardiologist who was independent of the clinical research team was designated to review deaths (due to any cause) and specifically cardiovascular events of interest. At a high level, such events of interest will include death of any cause, non-fatal acute myocardial infarction, non-fatal stroke, hospitalization due to unstable angina, hospitalization due to heart failure and coronary revascularization procedures (i.e. percutaneous coronary intervention, coronary artery bypass grafting). The revascularization procedures will not be considered to be major adverse events of interest but will be reviewed by the independent clinician to ensure that events of interest (e.g. acute myocardial infarction) have not been missed.

The clinician will review cases of interest to determine if they meet accepted diagnostic criteria. Causality assessments will not be made by the clinician, nor will the clinician possess governance authority. The cardiologist will be blinded regarding any information relating to the imaging measurements.

All deaths and pre-specified major adverse cardiovascular events (i.e. “MACE”-type events) will be prospectively collected by investigators and classified independently by the independent cardiologist. Details on these pre-specified events are listed in section 4.

As noted above, events of interest will be identified primarily by the investigator, who may use an eCRF checkbox to mark any event as a “CV event of interest”. The study was under regulatory review by the National Research Ethics Service and the National Waiting Times Board (NWTB) which is the Sponsor.

# Objective of the Event Adjudication Charter

The purpose of this document is to delineate the roles, responsibilities and procedures in regards to the adjudication of cardiovascular events occurring in the BHF MR-MI study.

## Study Coordinator

The independent cardiologist is assisted by the study coordinator (Dr David Carrick, BHF Cardiovascular Research Centre, University of Glasgow; [david.carrick@nhs.net](mailto:david.carrick@nhs.net)) who is a registered physician based in the University of Glasgow and Golden Jubilee National Hospital and who has considerable previous experience in the conduct of clinical cardiology studies.

The coordinator will:

Assist with preparation of the source clinical data

Enter the classification verdicts of the independent cardiologist into the database

# Events to be reviewed by the independent cardiologist

## 3.1 Deaths

The independent cardiologist will review all reported deaths and classify the cause of death according to the following schema:

Non-cardiovascular

A definite non-cardiovascular cause of death must be identified.

Cardiovascular (CV)

Death due to acute myocardial infarction

Death due to stroke

Sudden cardiac death

Other CV death (e.g. heart failure, pulmonary embolism, cardiovascular procedure-related)

Undetermined cause of death (i.e. cause of death unknown)

## 3.2 Non-fatal cardiovascular events

The independent cardiologist will review and adjudicate the following reported non-fatal cardiovascular events:

Acute myocardial infarction

Hospitalization for unstable angina/other angina*/chest pain*

Stroke/TIA/Other cerebrovascular events (i.e. subdural/extradural hemorrhage)**

Heart failure requiring hospitalization

Coronary revascularization procedures (i.e. percutaneous coronary intervention, coronary artery bypass grafting)***

Renal failure (>25% rise in creatinine from baseline or an absolute increase in serum creatinine of 0·.5 mg/dL (44 µmol/L) after a radiographic examination using a contrast agent (Barrett NEJM 2006;354:379-86)

Bleeding according to the ACUITY criteria (Stone Am Heart J 2004;148:764-75)

Note: Other non-fatal cardiovascular events will not routinely be reviewed by the independent cardiologist. These events will be reviewed by trained and qualified clinical research staff in the Golden Jubilee National Hospital to ensure that potential cardiovascular events requiring adjudication are not missed. If the review suggests that a potential cardiovascular event requiring adjudication may have been missed, further information will be requested, as required and, if necessary, the event will be allocated for adjudication.

*Hospitalization for other angina or for chest pain are not study events of interest but such events will be reviewed by the independent cardiologist to ensure that acute myocardial infarction or hospitalization for unstable angina events have not been missed.

**TIAs and other cerebrovascular events (subdural hemorrhage, extradural hemorrhage) will be reviewed to ensure that stroke events have not been missed.

***Coronary revascularization procedures (i.e. percutaneous coronary intervention, coronary artery bypass grafting) are not study events of interest but will be reviewed by the independent cardiologist to sure that study events of interest (e.g. acute myocardial infarction, hospitalization for unstable angina) have not been missed.

# Adverse Event definitions

For those event-types requiring adjudication, each event will usually be adjudicated on the basis of strict application of the endpoint definitions below. However, the clinical likelihood that a suspected event has occurred will be individually assessed even in the absence of fulfilment of all of the criteria specified in the event-definition, recognizing that information may at times be difficult to interpret (e.g. the exact measurement of ECG changes may be imprecise) or unavailable.

Overall, event definitions should align with the "Standardized definitions for endpoint events in cardiovascular trials' Hicks KA et al May 2011 and the "Third Universal Definition of Myocardial Infarction" Thygesen et al Eur Heart J 2012.

## 4.1 Deaths

In cases where a patient experiences an event and later dies due to that event, the event causing death and the death will be considered as separate events *only* if they are separated by a change in calendar day. If the event causing death and the death occur on the same calendar day, death will be the only event classified.

## 4.1.1 Cardiovascular deaths

**Cardiovascular death** includes death resulting from an acute myocardial infarction, sudden cardiac death, death due to heart failure, death due to stroke and death due to other cardiovascular causes as follows:

**Death due to Acute Myocardial Infarction** refers to a death usually occurring up to 30 days after a documented acute myocardial infarction (verified either by the diagnostic criteria outlined below for acute myocardial infarction, above, or by autopsy findings showing recent myocardial infarction or recent coronary thrombus) due to the myocardial infarction or its immediate consequences (e.g. progressive heart failure) and where there is no conclusive evidence of another cause of death.

If death occurs before biochemical confirmation of myocardial necrosis can be obtained, adjudication should be based on clinical presentation and other (e.g. ECG, angiographic, autopsy) evidence.

NOTE: This category will include sudden cardiac death, involving cardiac arrest, often with symptoms suggestive of myocardial ischemia, and accompanied by presumably new ST elevation*, or new left bundle branch block*, or evidence of fresh thrombus in a coronary artery by coronary angiography and/or at autopsy, but death occurring before blood samples could be obtained, or at a time before the appearance of cardiac biomarkers in the blood (i.e. myocardial infarction Type 3 – see section 4.2.1, below).

*If ECG tracings are not available for review, the independent cardiologist may adjudicate on the basis of reported new ECG changes that have been clearly documented in the case records or in the case report form.

Death resulting from a procedure to treat an acute myocardial infarction [percutaneous coronary intervention (PCI), coronary artery bypass graft surgery (CABG)], or to treat a complication resulting from acute myocardial infarction, should also be considered death due to acute myocardial infarction.

Death resulting from a procedure to treat myocardial ischemia (angina) or death due to an acute myocardial infarction that occurs as a direct consequence of a cardiovascular investigation/procedure/operation that was not undertaken to treat an acute myocardial infarction or its complications should be considered as a death due to other cardiovascular causes.

**Sudden Cardiac Death** refers to a death that occurs unexpectedly in a previously stable patient. The cause of death should not be due to another adjudicated cause (e.g. acute myocardial infarction Type 3 – see section 4.2.1 below).

The following deaths should be included.

a. Death witnessed and instantaneous without new or worsening symptoms

b. Death witnessed within 60 minutes of the onset of new or worsening symptoms unless a cause other than cardiac is obvious.

c. Death witnessed and attributed to an identified arrhythmia (e.g., captured on an ECG recording, witnessed on a monitor), or unwitnessed but found on implantable cardioverter-defibrillator review.

d. Death in patients resuscitated from cardiac arrest in the absence of pre-existing circulatory failure or other causes of death, including acute myocardial infarction, and who die (without identification of a non-cardiac etiology) within 72 hours or without gaining consciousness; similar patients who died during an attempted resuscitation.

Unwitnessed death without any other cause of death identified (information regarding the patient’s clinical status in the 24 hours preceding death should be provided, if available)

**Death due to Heart Failure** refers to a death occurring in the context of clinically worsening symptoms and/or signs of heart failure without evidence of another cause of death (e.g. acute myocardial infarction).

Death due to heart failure should include sudden death occurring during an admission for worsening heart failure as well as death from progressive heart failure or cardiogenic shock following implantation of a mechanical assist device.

New or worsening signs and/or symptoms of heart failure include any of the following:

**a**. New or increasing symptoms and/or signs of heart failure requiring the initiation of, or an increase in, treatment directed at heart failure or occurring in a patient already receiving maximal therapy for heart failure

Note: If time does not allow for the initiation of, or an increase in, treatment directed at heart failure or if the circumstances were such that doing so would have been inappropriate (e.g. patient refusal), the adjudication will be based on the clinical presentation and, if available, investigative evidence.

**b**. Heart failure symptoms or signs requiring continuous intravenous therapy (i.e. at least once daily bolus administration or continuous maintenance infusion) or chronic oxygen administration for hypoxia due to pulmonary edema.

**c**. Confinement to bed predominantly due to heart failure symptoms.

**d**. Pulmonary edema sufficient to cause tachypnea and distress **not** occurring in the context of an acute myocardial infarction, worsening renal function (that is not wholly explained by worsening heart failure/cardiac function) or as the consequence of an arrhythmia occurring in the absence of worsening heart failure.

**e**. Cardiogenic shock **not** occurring in the context of an acute myocardial infarction or as the consequence of an arrhythmia occurring in the absence of worsening heart failure.

Cardiogenic shock is defined as systolic blood pressure (SBP) < 90 mm Hg for greater than 1 hour, not responsive to fluid resuscitation and/or heart rate correction, and felt to be secondary to cardiac dysfunction and associated with at least one of the following signs of hypoperfusion:

Cool, clammy skin ***or***

Oliguria (urine output < 30 mL/hour) ***or***

Altered sensorium ***or***

Cardiac index < 2·2 L/min/m^2^

Cardiogenic shock can also be defined if SBP < 90 mm Hg and increases to ≥ 90 mm Hg in less than 1 hour with positive inotropic or vasopressor agents alone and/or with mechanical support.

**Death due to Stroke** refers to death after a documented stroke (verified by the diagnostic criteria outlined below for stroke or by typical post mortem findings) that is either a direct consequence of the stroke or a complication of the stroke and where there is no conclusive evidence of another cause of death.

NOTE: In cases of early death where confirmation of the diagnosis cannot be obtained, the independent may adjudicate based on clinical presentation alone.

Death due to a stroke reported to occur as a direct consequence of a cardiovascular investigation/procedure/operation will be classified as death due to other cardiovascular cause.

Death due to subdural or extradural hemorrhages will be adjudicated (based on clinical signs and symptoms as well as neuroimaging and/or autopsy) and classified separately by the CV-EAC.

**Death due to Other Cardiovascular Causes** refers to a cardiovascular death not included in the above categories [e.g. pulmonary embolism, cardiovascular intervention (other than one performed to treat an acute myocardial infarction or a complication of an acute myocardial infarction – see definition of death due to myocardial infarction, above), aortic aneurysm rupture, or peripheral arterial disease]. Mortal complications of cardiac surgery or non-surgical revascularization should be classified as cardiovascular deaths.

## 4.1.2 Non-cardiovascular deaths

A non-cardiovascular death is defined as any death that is not thought to be due to a cardiovascular cause. There should be unequivocal and documented evidence of a non-cardiovascular cause of death.

Further sub-classification of non-cardiovascular death will be as follows:

Pulmonary

Renal

Gastrointestinal

Infection (includes sepsis)

Non-infectious (e.g., systemic inflammatory response syndrome (SIRS))

Malignancy

Hemorrhage, not intracranial

Accidental/Trauma

Suicide

Non-cardiovascular surgery

Other non-cardiovascular, specify: ________________

## 4.1.3 Undetermined cause of death

This refers to any death not attributable to one of the above categories of cardiovascular death or to a non-cardiovascular cause (e.g. due to lack of information such as a case where the only information available is “patient died”). It is expected that every effort will be made to provide the adjudicating committee with enough information to attribute deaths to either a cardiovascular or non-cardiovascular cause so that the use of this category is kept to a minimal number of patients.

## 4.1.4 Non-fatal Cardiovascular Events

Date of onset

For purposes of classification, when classifying events that are a cause of hospitalization, the date of admission will be used as the onset date. In cases where the stated date of admission differs from the date the patient first presented to hospital with the event (e.g. because of a period of observation in an emergency department, medical assessment unit or equivalent), the date of initial presentation to hospital will be used (provided that the patient had not been discharged from hospital in the interim).

For events where an admission date is not applicable (or not available), the date of onset as stated by the investigator will be used.

## 4.2.1 Acute myocardial infarction

Note on biomarker elevations:

For cardiac biomarkers, laboratories should report an upper reference limit (URL). If the 99th percentile of the upper reference limit (URL) from the respective laboratory performing the assay is not available, then the URL for myocardial necrosis from the laboratory should be used. If the 99th percentile of the URL or the URL for myocardial necrosis is not available, the MI decision limit for the particular laboratory should be used as the URL.

## Spontaneous acute myocardial infarction:

A rise and/or fall of cardiac biomarkers (troponin or CK-MB) should usually be detected (see note below) with at least one value above the upper reference limit (URL) together with evidence of myocardial ischemia with at least one of the following:

- Clinical presentation consistent with ischemia
- ECG evidence of acute myocardial ischemia (as outlined in Table 1, below) or new left bundle branch block (LBBB).
- Development of pathological Q waves on the ECG (see Table 2, below)
- Imaging evidence of new loss of viable myocardium or new regional wall motion abnormality
- Autopsy evidence of acute myocardial infarction
- If biomarkers are elevated from a prior infarction, then a spontaneous myocardial infarction is defined as:

**a**. One of the following:

o Clinical presentation consistent with ischemia

o ECG evidence of acute myocardial ischemia (as outlined in Table 1, below) or new left bundle branch block. [The events committee will adjudicate in the context of the sequential ECG changes that are commonly seen in acute ST elevation/acute non-ST elevation myocardial infarction.]

o New pathological Q waves (see Table 2, below). [The events committee will adjudicate in the context of the sequential ECG changes that are commonly seen in acute ST elevation/acute non-ST elevation myocardial infarction.]

o Imaging evidence of new loss of viable myocardium or new regional wall motion abnormality

o Autopsy evidence of acute myocardial infarction

AND

**b**. Both of the following:

o Evidence that cardiac biomarker values were decreasing (e.g. two samples 3-6 hours apart) prior to the suspected acute myocardial infarction*

o ≥ 20% increase (and > URL) in troponin or CK-MB between a measurement made at the time of the initial presentation with the suspected recurrent myocardial infarction and a further sample taken 3-6 hours later

- *If biomarkers are increasing or peak is not reached, then a definite diagnosis of recurrent myocardial infarction is generally not possible.

Percutaneous coronary intervention-related acute myocardial infarction

Peri-percutaneous coronary intervention (PCI) acute myocardial infarction is defined by any of the following criteria. Symptoms of cardiac ischemia are not required.

Biomarker elevations within 48 hours of PCI:

• Troponin or CK-MB (preferred) > 5 x URL ***and***

• No evidence that cardiac biomarkers were elevated prior to the procedure;

OR

• Both of the following must be true:

o ≥ 50% increase in the cardiac biomarker result

o Evidence that cardiac biomarker values were decreasing (e.g. two samples 3-6 hours apart) prior to the suspected acute myocardial infarction

New pathological Q waves or new left bundle branch block (LBBB).

[If the PCI was undertaken in the context of an acute myocardial infarction, the events committee will adjudicate in the context of the sequential ECG changes that are commonly seen in acute ST elevation/acute non-ST elevation myocardial infarction.]

Autopsy evidence of acute myocardial infarction

Coronary artery bypass grafting-related acute myocardial infarction

Peri-coronary artery bypass graft surgery (CABG) acute myocardial infarction is defined by the following criteria. Symptoms of cardiac ischemia are not required.

Biomarker elevations within 72 hours of CABG:

• Troponin or CK-MB (preferred) > 10 x URL ***and***

• No evidence that cardiac biomarkers were elevated prior to the procedure;

OR

• Both of the following must be true:

o ≥ 50% increase in the cardiac biomarker result

o Evidence that cardiac biomarker values were decreasing (e.g. two samples 3-6 hours apart) prior to the suspected acute myocardial infarction

AND

One of the following:

New pathological Q-waves (preferably with evidence of persistence)

[If the CABG was undertaken in the context of an acute myocardial infarction, the events committee will adjudicate in the context of the sequential ECG changes that are commonly seen in acute ST elevation/acute non-ST elevation myocardial infarction.]

New LBBB (preferably with evidence of persistence)

Angiographically documented new graft or native coronary artery occlusion

Imaging evidence of new loss of viable myocardium

OR

Autopsy evidence of acute myocardial infarction

**Note:** For a diagnosis of acute myocardial infarction, a rise and/or fall of cardiac biomarkers should usually be detected. However, myocardial infarction may be adjudicated for an event that has characteristics which are very suggestive of acute infarction but which does not meet the strict definition because biomarkers are not available (e.g. not measured) or are non-contributory (e.g. may have normalized).

Suggestive characteristics are:

Typical cardiac ischemic-type pain/discomfort
(except for suspected acute myocardial infarction occurring in the context of PCI or CABG where this requirement need not apply)

AND

New ECG changes* or other evidence to support a diagnosis of acute myocardial infarction (e.g. imaging evidence of new loss of viable myocardium/new regional wall motion abnormality or angiography demonstrating occlusive coronary thrombus)

*If ECG tracings are not available for review, the adjudication may be made on the basis of reported ECG changes that have been clearly documented in the case records or in the case report form.

Clinical classification of different types of myocardial infarction

Myocardial infarctions will be clinically classified as:

Type 1

Spontaneous myocardial infarction related to ischemia due to a primary coronary event such as plaque erosion and/or rupture, fissuring, or dissection.

Type 2

Myocardial infarction secondary to ischemia due to either increased oxygen demand or decreased supply, e.g. coronary artery spasm, coronary embolism, anemia, arrhythmias, hypertension, or hypotension.

Type 3

Sudden unexpected cardiac death, including cardiac arrest, often with symptoms suggestive of myocardial ischemia, accompanied by presumably new ST elevation, or new LBBB, or evidence of fresh thrombus in a coronary artery by angiography and/or at autopsy, but death occurring before blood samples could be obtained, or at a time before the appearance of cardiac biomarkers in the blood.

Type 4a

Myocardial infarction associated with PCI.

Type 4b

Myocardial infarction associated with stent thrombosis as documented by angiography or at autopsy.

Type 5

Myocardial infarction associated with CABG.

Myocardial infarctions will be further sub-classified as:

ST segment elevation myocardial infarction (STEMI).
**or**

Non-ST segment elevation myocardial infarction (NSTEMI).
**or**

Myocardial infarction, type (i.e. STEMI or NSTEMI) unknown.

| Table 1: ECG manifestations of acute myocardial ischemia (in absence of left ventricular hypertrophy and left bundle branch block) |
| --- |
| ST elevation  New ST elevation at the J-point in two anatomically contiguous leads with the cut-off  points: ≥ 0·2 mV in men (> 0·25 mV in men < 40 years) or ≥ 0·15 mV in women in leads V2-V3 and/or ≥ 0·1 mV in other leads.  ST depression and T wave changes  New horizontal or down-sloping ST depression ≥ 0·05 mV in two  contiguous leads; and/or new T wave inversion ≥ 0·1 mV in two contiguous  leads.  The above ECG criteria illustrate patterns consistent with myocardial ischemia. In patients with abnormal biomarkers, it is recognized that lesser ECG abnormalities may represent an ischemic response and may be accepted under the category of abnormal ECG findings. |

| Table 2: Pathological Q waves: |
| --- |
| Any Q-wave in leads V2-V3 ≥ 0·02 seconds or QS complex in leads V2 and V3  Q-wave ≥ 0·03 seconds and ≥ 0·1 mV deep or QS complex in leads I, II, aVL, aVF, or V4-V6 in any two leads of a contiguous lead grouping (I, aVL, V6; V4-V6; II, III, and aVF) a  A The same criteria are used for supplemental leads V7-V9, and for the Cabrera frontal plane lead grouping. |

## 4.2.2 Hospitalization for unstable angina

For the diagnosis of hospitalization due to unstable angina there should be emergency/unplanned admission to a hospital setting (emergency room, observation or inpatient unit) that results in at least one overnight stay (i.e. a date change) with fulfilment of the following criteria:

There should be:

1. Cardiac ischemic-type symptoms at rest (chest pain or equivalent) or an accelerating pattern of angina (e.g. exercise-related ischemic-type symptoms increasing in frequency and/or severity, decreasing threshold for onset of exercise related ischemic type symptoms) but without the fulfilment of the above diagnostic criteria for acute myocardial infarction.

and

2 The need for treatment with parenteral (intravenous, intra-arterial, buccal, transcutaneous or subcutaneous) anti-ischemic/antithrombotic therapy and/or coronary revascularization.

and

3a ECG manifestations of acute myocardial ischemia (New ST-T changes meeting the criteria for acute myocardial ischemia - as outlined in Table 1, section 5.2.1).

or

3b Angiographically significant coronary artery disease thought to be responsible for the patient’s presentation. [If both invasive and CT angiographic imaging of the coronary arteries were performed, the results of the invasive coronary angiogram should take preference.]

and

4 The independent clinician should be satisfied that unstable angina was the primary reason for hospitalization.

## 4.2.3 Hospitalization for other angina*

For the diagnosis of hospitalization for other angina, there should be emergency/unplanned admission to a hospital setting (emergency room, observation or inpatient unit) that results in at least one overnight stay (i.e. a date change) with fulfilment of the following criteria:

There should be:

Typical cardiac ischemic-type symptoms but without the fulfilment of the above diagnostic criteria for acute myocardial infarction or unstable angina.

and

2 The need for treatment with new or increased anti-anginal therapy (excluding sublingual nitrate therapy).

and

3a Investigations undertaken in view of the event (e.g. exercise ECG or stress myocardial perfusion scan) showing evidence of reversible myocardial ischemia.

or

3b Coronary angiography showing angiographically significant coronary disease thought to be responsible for the patient’s presentation. [If both invasive and CT angiographic imaging of the coronary arteries were performed, the results of the invasive coronary angiogram should take preference.]

and

4 The independent clinician should be satisfied that angina was the primary reason for hospitalization.

## 4.2.4 Hospitalization for other chest pain*

There should be:

Emergency/unplanned admission to a hospital setting (emergency room, observation or inpatient unit) that results in at least one overnight stay i.e. a date change) due to chest pain but where the definitions (above) of acute myocardial infarction, hospitalization for unstable angina or hospitalization for other angina are not met.

The independent clinician should be satisfied that chest pain was the primary reason for hospitalization.

*These events are not study cardiovascular events of interest but the definitions provided for these events will be used by the independent clinician to categorize reported myocardial infarction, angina and chest pain events that do not meet the study definition of acute myocardial infarction or hospitalization for unstable angina.

## 4.2.5 Stroke

**Stroke** is defined as an acute episode of neurological dysfunction caused by focal or global brain, spinal cord, or retinal vascular injury.

**A** For the diagnosis of stroke, the following 4 criteria should usually be fulfilled:

1. Rapid onset* of a focal/global neurological deficit with at least one of the following:

Change in level of consciousness

Hemiplegia

Hemiparesis

Numbness or sensory loss affecting one side of the body

Dysphasia/aphasia

Hemianopia (loss of half of the field of vision of one or both eyes)

Complete/partial loss of vision of one eye

Other new neurological sign(s)/symptom(s) consistent with stroke

*If the mode of onset is uncertain, a diagnosis of stroke may be made provided that there is no plausible non-stroke cause for the clinical presentation.

2. Duration of a focal/global neurological deficit > 24 hours

or

< 24 hours if

(i) this is because of at least one of the following therapeutic interventions:

(a) pharmacologic i.e. thrombolytic drug administration.

(b) non-pharmacologic i.e. neurointerventional procedure (e.g. intracranial angioplasty).

or

(ii) brain imaging available clearly documenting a new hemorrhage or infarct.

or

(iii) the neurological deficit results in death

3. No other readily identifiable non-stroke cause for the clinical presentation (e.g. brain tumor, hypoglycemia, peripheral lesion).

4. Confirmation of the diagnosis by at least one of the following**:

neurology or neurosurgical specialist.

brain imaging procedure (at least one of the following):

CT scan.

MRI scan.

cerebral vessel angiography.

lumbar puncture (i.e. spinal fluid analysis diagnostic of intracranial hemorrhage).

B If the acute neurological deficit represents a worsening of a previous deficit, this worsened deficit must have:

Persisted for more than one week

**Or** < one week if

(i) this is because of at least one of the following therapeutic interventions:

(a) pharmacologic i.e. thrombolytic drug administration.

(b) non-pharmacologic i.e. neurointerventional procedure (e.g. intracranial angioplasty).

or

(ii) brain imaging available clearly documenting an appropriate new CT/MRI finding.

or

(iii) the neurological deficit results in death

Strokes will be further sub-classified as:

Ischemic (non-hemorrhagic) stroke

(i.e. caused by an infarction of central nervous system tissue)

or

Hemorrhagic stroke***

(i.e. caused by nontraumatic intraparenchymal, intraventricular or subarachnoid hemorrhage)

or

Stroke type (i.e. hemorrhagic or ischemic) unknown (i.e. when imaging/other investigations are unavailable or inconclusive).

***Subdural and extradural hemorrhages will be adjudicated (based on clinical signs and symptoms as well as neuroimaging and/or autopsy) and classified separately.

## 4.2.6. Heart Failure requiring hospitalization

For the diagnosis of heart failure requiring hospitalization, there should be emergency/unplanned admission to a hospital setting (emergency room, observation or inpatient unit) that results in at least one overnight stay (i.e. a date change) with fulfilment of the following criteria:

There should be:

Clinical manifestations of new or worsening heart failure including at least one of the following:

New or worsening dyspnea on exertion

New or worsening dyspnea at rest

New or worsening fatigue/decreased exercise tolerance

New or worsening orthopnea

New or worsening PND (paroxysmal nocturnal dyspnea)

New or worsening lower limb or sacral edema

New or worsening pulmonary crackles/crepitations

New or worsening elevation of JVP (jugular venous pressure)

New or worsening third heart sound or gallop rhythm

**And**

1. Investigative evidence of structural or functional heart disease (if available) with at least *one* of the following:

Radiological evidence of pulmonary edema/congestion or cardiomegaly.

Imaging ( e.g. echocardiography, cardiac magnetic resonance imaging, radionuclide ventriculography) evidence of an abnormality (e.g. left ventricular systolic dysfunction, significant valvular heart disease, left ventricular hypertrophy).

- - Elevation of BNP or NT-proBNP levels.
  - Other investigative evidence of structural or functional heart disease (e.g. evidence obtained from pulmonary artery catheterization).

**And**

**3** Need for new/increased therapy***** specifically for the treatment of heart failure

including at least one of the following:

New or increased oral therapy for the treatment of heart failure

(See note on oral therapy, below)

Initiation of intravenous diuretic, inotrope, vasodilator or other recognized intravenous heart failure treatment or uptitration of such intravenous therapy if already receiving it

Mechanical or surgical intervention (e.g. mechanical or non-invasive ventilation, mechanical circulatory support, heart transplantation, ventricular pacing to improve cardiac function), or the use of ultrafiltration, hemofiltration, dialysis or other mechanical or surgical intervention that is specifically directed at treatment of heart failure.

Note on oral therapy: In general, for an event to qualify as *heart failure requiring hospitalization* on the basis of *oral* heart failure therapy (i.e. in cases where none of the non-pharmacological treatment modalities listed above have been utilized), the new or increased oral therapy should include oral diuretics. However, in special cases, other new or increased oral therapy (e.g. hydralazine/long acting nitrate, aldosterone antagonist) may be accepted provided that the adjudication committee is satisfied that:

the new or increased oral therapy was primarily directed at treating clinical manifestations of new or worsening heart failure (rather than, for example, initiation or up-titration of heart failure therapy as part of the routine optimization of medical therapy)

and

the totality of the evidence indicates that heart failure, rather than any other disease process, was the primary cause of the clinical presentation.

*****If time does not allow for the initiation of, or an increase in, treatment directed at heart failure or if the circumstances were such that doing so would have been inappropriate (e.g. patient refusal), the independent clinician will adjudicate on clinical presentation and, if available, investigative evidence.

and

**4** The independent clinician should be satisfied that heart failure was the primary disease process accounting for the clinical presentation.

## 4.2.7. Renal Failure requiring hospitalization

Contrast-induced nephropathy: is defined as either a greater than 25% increase of serum creatinine or an absolute increase in serum creatinine of 0·5 mg/dL after a radiographic examination using a contrast agent.

## 4.2.8. Bleeding requiring hospitalization

Bleeding: is defined according to the ACUITY criteria: major bleed = intracranial or intraocular bleeding; bleeding at the site of angiography requiring intervention; a hematoma of 5 cm in diameter; a reduction in hemoglobin level of at least 4 g/dL in the absence of overt bleeding or 3 g/dL with a source of bleeding; or transfusion.

## 6.1 Event identification

The BHF MR-MI study will use paper-based and electronic data capture (EDC). Those events requiring independent validation (see section 4) will be reported by the Investigator via the EDC (electronic data capture) system.

## 6.2 Incomplete event data

If, having reviewed the event data pertaining to an event, the independent cardiologist deems that the information provided is insufficient for the purposes of event adjudication, an electronic request for further information detailing the information required will be made. The date of request will be recorded electronically and the event will be classified as not adjudicated/pending additional information.

# Clinical data to be provided

The trial management team (including Prof Berry, Dr Carrick, Ms Joanne Kelly CRN) will provide event data for each potential cardiovascular event requiring adjudication to the independent cardiologist.

Data to be included for event classification will include:

Subject study identification number and event details

On request: Relevant de-identified CRF data (including any relevant event-specific CRFs e.g. the *myocardial infarction/hospitalization for unstable angina/other angina/*

*chest pain* event form).

Supportive source documentation as required

Baseline and subsequent scheduled ECGs obtained during study participation.

All clinical data would be de-identified.

De-identified Source Documentation

The following source documents (if available) will be provided to the independent cardiologist as part of the standard dossier contents for cardiovascular events requiring review/adjudication:

Death

Hospital Discharge Summary/Death Summary

Autopsy Report

Death Certificate

Admission History & Physical (if applicable)

Acute Myocardial Infarction/Hospitalization for Unstable Angina/Other Angina/Chest Pain

Hospital Discharge Summary

ECGs

Pre-Randomization/Screening

Baseline (prior to event but post-randomization)

During Event

Post-Event

Relevant Procedure/Operation Reports

Relevant Laboratory Reports (e.g. that document the cardiac enzyme/marker measurements provided – peak values and pre-procedure and post-procedure values, where applicable)

Reports for other investigations taken:

PCI Report

CABG Report

Coronary Angiography Report

Echocardiogram Report

Exercise ECG Report

Stress Myocardial Perfusion Scan Report

Other investigation report undertaken to test for presence of reversible myocardial ischemia

Admission History & Physical

Stroke/TIA/Other cerebrovascular events

Hospital Discharge Summary

Neurology Consultation Report(s)

Reports for other investigations undertaken:

CT Brain Scan Report

MRI Brain Scan Report

Cerebral Angiography Report

Lumbar Puncture Report

Admission History & Physical

Heart Failure requiring hospitalization

Hospital Discharge Summary

Chest X-Ray Report

Prescription Sheets/Medication Administration Records

Echocardiogram Report

Relevant Laboratory Reports (e.g. for peak BNP/NT-proBNP)

Reports for other investigations undertaken:

Cardiac Magnetic Resonance Imaging

Radionuclide Ventriculogram Scan

Pulmonary Artery Catheterization

Admission History & Physical

Coronary revascularization procedure

Hospital Discharge Summary

Relevant Procedure/Operation Reports

Bleeding

Hospital Discharge Summary

Relevant Procedure/Operation Reports

Hb

Blood transfusion results

Diagnostic and therapeutic procedures (e.g. gastroscopy).

**Supplementary Results**

**Myocardial native T1 according to infarct-artery territory**

Culprit artery territory was designated according to the American Heart Association model [38], individualized for each patient according to coronary artery dominance.

The mean myocardial T1 values of remote and injured regions by culprit artery territory were similar: left anterior descending artery vs. left circumflex vs. right coronary artery (963.9±23.7 ms and 1088.7±48.4 ms vs. 965.6±26.9 ms and 1105.1±57.7 ms vs. 957.5±25.1 ms and 1104.1±51.3 ms, respectively; p=0.059 and p=0.084).

**Myocardial native T1 in STEMI patients and healthy volunteers**

Fifty healthy volunteers from the same geographical region (52% male, mean (SD) age 54 (13) years) without a history of cardiovascular disease or therapy were enrolled during the same time period as the STEMI patients. The volunteers were scanned using the same 1.5 Tesla MRI scanner (Siemens AVANTO) as the STEMI patients, and the approach to image analysis was the same as for STEMI patients also, including regional segmentation of the left ventricle according to the American Heart Association model [38].

At the mid-ventricular level, mean remote zone native T1 was similar in STEMI patients (961 (25) ms) and healthy volunteers (958 (24); p=0.314). Remote zone native T1 was higher in male STEMI patients than in male volunteers (959 (25) vs. 948 (20) ms, respectively; p=0.024), but similar in female STEMI patients (968 (25) ms) and volunteers (968 (23) ms). In healthy subjects, mid-ventricular T1 values were lower in males than in females (948 (20) ms vs. 968 (23) ms; p=0.003). In both men and women, the infero-lateral segment had the highest T1 compared to the antero-septal segment (960 (28) ms vs. 939 (26) ms and 978 (32) ms vs. 961 (34) ms, respectively; p<0.001 and p=0.011).

The coefficients of variation (CoV) for native T1 in the mid-ventricular level with regions-of-interest within myocardial regions were: anterior segment CoV = 2.35; antero-lateral segment CoV = 2.98; antero-septal segment CoV = 3.35; inferior segment CoV = 2.49; infero-lateral segment CoV = 3.22; infero-septal segment CoV = 2.90.

**Intra- and inter-observer agreement of T1 measurements**

Native T1 in regions-of-interest in remote and injured zones in a subgroup of 20 randomly chosen patients were independently measured by two observers. The intra-class correlation coefficient for reliability of remote T1 was 0.92 (95% confidence interval (CI): 0.80, 0.97); p<0.001). Bland-Altman plots showed no evidence of bias. The coefficient of variation for remote zone native T1 was 2.61.

**Univariable correlates of LV end-diastolic volume at 6 months**

The clinical characteristics that were univariably associated with LV end-diastolic volume at 6 months that were also included in the multivariable model were age (p=0.338), previous myocardial infarction (p=0.820), diabetes mellitus (p=0.071), previous percutaneous coronary intervention (p=0.859), cigarette smoking (p=0.397), history of hypertension (p=0.112), history of angina (p=0.688), heart rate (p=0.119), systolic blood pressure at initial angiography (p=0.097), Killip class II vs. Killip class I (reference category) (p=0.974), Killip class III vs. Killip class I (reference category) (p=0.860), Killip class IV vs. Killip class I (reference category) (p=0.122), symptom onset to reperfusion time (p=0.602), TIMI flow grade 2 vs. grade 1 (reference category) at initial angiography (p=0.188), TIMI flow grade 3 vs. grade 1 (reference category) at initial angiography (p=0.352), TIMI flow grade 2 vs. grade 1 (reference category) at the end of PCI (p=0.695), TIMI flow grade 3 vs. grade 0/1 (reference category) at the end of PCI (p=0.497), ST segment resolution (none vs. complete (reference category), p=0.052; incomplete vs. complete (reference category), p=0.199), reperfusion mode (failed vs. primary PCI, p=0.096).

The multivariable correlates of LV end-diastolic volume at 6 months in 267 patients with follow-up CMR are described in Table 4 in the main paper.

**Net reclassification index for remote zone native T1 and prognostic models**

The net reclassification index for adding remote zone native T1 to these models was p>0.05 in all cases, indicating that adding remote zone native T1 neither significantly improved nor worsened the predicted probability of the event. The C-index for MACE post-discharge and T1 (unadjusted) was 0.583 and 0.577 and 0.590 after adjustment for baseline LVEDV and change in CRP, respectively.

**Receiver operating statistics for remote zone native T1 and all adverse cardiovascular outcomes**

In an exploratory analysis instigated based on peer review, we assessed the relationships between remote zone native T1 values and all adverse cardiac outcomes in the following composite: *all-cause death and heart failure, major adverse cardiovascular events, major adverse cardiac and cerebrovascular events (including stroke and transient ischemic attack), and the occurrence of adverse LV remodelling (surrogate adverse outcome).* In this way the number of adverse outcomes and power is increased.

*a) T1 remote zone cut-point of 963.25 (ms)*

| Remote zone native T1 cut-off, ms | < 963.25 | ≥ 963.25 |
| --- | --- | --- |
| No event | 128 | 106 |
| Event | 24 | 30 |

Sensitivity: 0.56; Specificity: 0.55; positive predictive value: 0.22; negative predictive value: 0.84

*b)      T1 remote zone cut-point of 965.60 ms*

| Remote zone native T1 cut-off, ms | < 965.60 | ≥ 965.60 |
| --- | --- | --- |
| No event | 141 | 93 |
| Event | 25 | 29 |

 Sensitivity: 0.54; specificity: 0.60; positive predictive value: 0.24; negative predictive value: 0.85

*c)       Upper quartile as cut-point: 969 ms*

| Remote zone native T1 cut-off, ms | < 969 | ≥ 969 |
| --- | --- | --- |
| No event | 160 | 74 |
| Event | 31 | 23 |

Sensitivity: 0.43; specificity: 0.69; positive predictive value: 0.24; negative predictive value: 0.84.

**Supplementary Table 1.** Clinical characteristics of 288 STEMI patients who had CMR 2 days post-MI with evaluable myocardial native T1 maps. The patients are grouped according to the availability of CMR at 6 months (n=267 (93%)) or not (n=21 (7%)). The selected characteristics that are listed in this table differed between patients who had a 6 months follow-up CMR scan or not.

| Characteristics* | All patients | CMR at 6 months | No CMR at 6 months | P-value |
| --- | --- | --- | --- | --- |
|  | n=288 | n = 267 (93%) | n = 21 (7%) |  |
| *Clinical characteristics* |  |  |  |  |
| Male sex, n (%) | 211 (73) | 197 (74) | 14 (67) | 0.011 |
| ECG |  |  |  |  |
| ST segment elevation resolution post PCI, n (%) |  |  |  |  |
| Complete, ≥70 % | 129 (45) | 122 (46) | 7 (33) |  |
| Incomplete, 30% to < 70% | 115 (40) | 102 (38) | 13 (62) | 0.036 |
| None, ≤30% | 43 (15) | 42 (16) | 1 (5) |  |
| *Initial blood results on admission* |  |  |  |  |
| C-reactive protein, (mg/L) | 3.0 (2.0 - 7.0)  0 - 265.0 | 3.0 (2.0 - 7.0)  0 - 265.0 | 4.0 (2.0 - 12.2)  1.0 - 92.0 | 0.034 |
| Leucocyte cell count (x10^9^L) | 12.4 (3.5) | 12.3 (3.6) | 13.1 (2.5) | 0.002 |
| Neutrophil count (x10^9^L) | 9.6 (3.2) | 9.5 (3.3) | 10.4 (2.2) | 0.002 |
| *CMR findings* |  |  |  |  |
| LV ejection fraction, % | 55 (10) | 55 (10) | 51 (8) | 0.041 |
| LV end-diastolic volume, ml |  |  |  |  |
| Men | 162 (33) | 161 (32) | 170 (44) | 0.017 |
| LV end-systolic volume, ml |  |  |  |  |
| Men | 76 (26) | 75 (26) | 84 (35) | <0.001 |
| Area at risk, % LV mass | 32 (12) | 32 (12) | 33 (15) | 0.045 |
| Infarct size, % LV mass | 18 (13) | 17 (13) | 23 (17) | 0.044 |
| Late microvascular obstruction, % LV mass | 2.7 (4.6) | 2.5 (4.2) | 5.4 (7.7) | 0.021 |
| T1 remote myocardium (all subjects), ms | 961 (25) | 961 (26) | 963 (16) | <0.001 |

Footnote: ECG = electrocardiogram. All of the other clinical characteristics (Table 1) and CMR findings (Table 2) were similar (p>0.05) for patients according to the availability of a CMR scan at 6 months (n=267) or not (n=21).

**Supplementary Table 2.** Blood results from acute 288 STEMI patients obtained from samples taken initially on arrival in hospital and then the peak value obtained during subsequent testing within 48 hours and the change from the admission result.

| Blood results |  | All STEMI patients  n=288 |
| --- | --- | --- |
| *Initial result on admission* |  |  |
| C-reactive protein, (mg/L) | median (IQR)  range, g/L | 3.0 (2.0, 7.0)  0 - 265.0 |
| Leucocyte cell count (x10^9^L) |  | 12.36 (3.48) |
| Neutrophil count (x10^9^L) |  | 9.56 (3.24) |
| Monocytes (x10^9^L) |  | 0.84 (0.36) |
| Eosinophils (x10^9^L) |  | 0.12 (0.13) |
| Lymphocytes (x10^9^L) |  | 1.79 (0.79) |
| Basophils (x10^9^L) |  | 0.03 (0.02) |
| *Maximum result after admission* |  |  |
| C-reactive protein, (mg/L) | median (IQR)  range, g/L | 8.0 (4.0, 16.0)  0 - 347.0 |
| Leucocyte cell count (x10^9^L) |  | 12.8 (3.39) |
| Neutrophil count (x10^9^L) |  | 9.86 (3.16) |
| Monocytes (x10^9^L) |  | 1.08 (0.38) |
| Eosinophils (x10^9^L) |  | 0.17 (0.15) |
| Lymphocytes (x10^9^L) |  | 2.31 (0.83) |
| Basophils (x10^9^L) |  | 0.03 (0.02) |
| *Change within 2 days from admission* |  |  |
| C-reactive protein, (mg/L) | median (IQR)  range, g/L | 3.0 (0.0, 9.0)  0 - 266.0 |
| Leucocyte cell count (x10^9^L) |  | 2.53 (2.02) |
| Neutrophil count (x10^9^L) |  | 2.87 (2.14) |
| Monocytes (x10^9^L) |  | 0.31 (0.25) |
| Eosinophils (x10^9^L) |  | 0.08 (0.08) |
| Lymphocytes (x10^9^L) |  | 0.72 (0.53) |
| Basophils (x10^9^L) |  | 0.01 (0.01) |

Reference range in SI units: C-reactive protein (mg/L) < 10; leucocyte count (x10^9^/L) 4.0-11.0 (x10^9^/L); neutrophil count (x10^9^/L) 2.0 –7.5; lymphocyte count (x10^9^/L) 1.50 – 4.0; monocytes (x10^9^/L) 0.2 - 0.80; eosinophils (x10^9^/L) 0.04 - 0.40; basophils (x10^9^/L) 0.02 - 0.10.

**Supplementary Table 3.** Associations of patient characteristics with native T1 (ms) in remote myocardium in univariable regression analyses (n=288).

| Associations | Coefficient (95% CI) | P-value |
| --- | --- | --- |
| *Univariable*  *Patient characteristics and angiographic findings* | |  |
| Male sex | -9.73 (-16.21, -3.25) | 0.003 |
| Previous myocardial infarction | 12.22 (1.57, 22.86) | 0.025 |
| Killip class IV | 37.69 (9.39, 66.00) | 0.009 |
| No ST-segment resolution | 13.25 (4.73, 21.78) | 0.002 |
| Incomplete ST-segment resolution | 9.67 (3.46, 15.87) | 0.002 |
| *CRP and leucocytes* |  |  |
| Initial log CRP, mg/L | 3.79 (1.28, 6.29) | 0.003 |
| Peak log CRP, mg/L | 3.35 (0.95, 5.75) | 0.006 |
| Peak monocyte count, x10^9^L | 7.37 (-0.22, 14.96) | 0.057 |
| *CMR findings at baseline* |  |  |
| Area-at-risk on T2 map, % LV mass | 0.38 (0.13, 0.62) | 0.002 |
| LV ejection fraction, % | -0.40 (-0.70, -0.10) | 0.009 |
| LV end-systolic volume, ml | 0.11 (-0.00, 0.22) | 0.058 |
| Infarct size, % LV mass | 0.31 (0.09, 0.52) | 0.006 |
|  |  |  |

For univariable analyses, all variables in Table 1 were tested and also the following baseline CMR parameters: area-at-risk, LV ejection fraction, LV end-diastolic volume, LV end-systolic volume and infarct size.

**Supplementary Table 4.** Univariable and multivariable predictors of left ventricular end-diastolic volume at 6 months post-STEMI in 121 STEMI patients without microvascular obstruction at baseline.

| Multivariable associations | coefficient (95% CI) | p value |
| --- | --- | --- |
| *Patient characteristics, angiographic findings and initial infarct size*† | |  |
| **Infarct zone native T1, ms** | **0.09 (0.02, 0.16)** | **0.003** |
| LV end-diastolic volume at baseline, ml | 0.69 (0.56, 0.82) | <0.001 |
| Infarct size at baseline, % left ventricular mass | 0.54 (0.07, 1.01) | 0.025 |
| Male sex | 12.89 (4.87, 20.91) | 0.002 |
| Diabetes mellitus | -13.68 (-25.70, -1.65) | 0.026 |
| Hypercholesterolaemia | -11.87 (-19.70, -4.03) | 0.003 |
| Systolic blood pressure at initial angiography, mmHg | 0.16 (0.04, 0.28) | 0.011 |
| Killip class IV | 102.52 (66.41, 138.63) | <0.001 |
| Reperfusion mode (successful thrombolysis) | 57.33 (22.53, 92.13) | 0.002 |

Footnote: The coefficient (95% confidence intervals) indicates the magnitude and direction of the difference in left ventricular end-diastolic volume (ml) at follow-up for the patient characteristic (binary or continuous). For example, on average, left ventricular end-diastolic volume (ml) at follow-up is 0.69 (0.56, 0.82) higher for each 1 ms increase in remote zone native T1 measured by CMR at baseline.

*The clinical characteristics that were univariable predictors of left ventricular end-diastolic volume that were also included in the multivariable model were age (p=0.238), body mass index (p=0.189), previous myocardial infarction (p=0.624), previous percutaneous coronary intervention (p=0.925), cigarette smoking (p=0.313), history of hypertension (p=0.218), history of angina (p=0.657), heart rate (p=0.131), Killip class II vs. Killip class I (reference category) (p=0.087), Killip class III vs. Killip class I (reference category) (p=0.825), sustained ventricular arrhythmia (p=0.086), symptom onset to reperfusion time (p=0.309), TIMI flow grade 2 vs. grade 1 (reference category) at initial angiography (p=0.379), TIMI flow grade 3 vs. grade 1 (reference category) at initial angiography (p=0.886), TIMI flow grade 2 vs. grade 1 (reference category) at the end of PCI (p=0.886), TIMI flow grade 3 vs. grade 0/1 (reference category) at the end of PCI (p=0.883), ST segment resolution at the end of PCI (none) (p=0.209), ST segment resolution at the end of PCI (partial) (p=0.059), reperfusion mode (failed vs. primary PCI, p=0.391).

†When area-at-risk was included instead of infarct size, remote zone T1 was also a predictor of left ventricular end-diastolic volume at follow-up (0.12 (0.05, 0.19); p=0.002).

Similar findings were also observed for the predictors of change in LV end-diastolic volume from baseline in patients without MVO (data not shown).

**Supplementary Table 5.** Univariable and multivariable predictors of change in left ventricular end-diastolic volume at 6 months post-STEMI compared with to baseline in 267 STEMI patients.

| Multivariable associations | coefficient (95% CI) | p value |
| --- | --- | --- |
| *Patient characteristics** | |  |
| **Remote zone native T1, ms** | **0.15 (0.02, 0.27)** | **0.021** |
| Male sex | 10.75 (3.64, 17.86) | 0.003 |
| Killip class III | 18.86 (5.22, 32.49) | 0.007 |
| TIMI flow grade 3 at initial angiography | -13.93 (-24.82, -3.04) | 0.012 |
| Reperfusion mode: successful thrombolysis | 63.06 (14.31, 111.81) | 0.011 |
| *Patient characteristics, angiographic findings and initial infarct size*† | |  |
| **Remote zone native T1, ms** | **0.13 (0.01, 0.25)** | **0.031** |
| Infarct size, % left ventricular mass | 0.70 (0.40, 0.99) | 0.006 |
| Male sex | 8.34 (1.47, 15.21) | 0.018 |
| History of hypertension | 7.08 (0.17, 14.00) | 0.045 |
| History of hypercholesterolemia | -7.50 (-14.85, -0.15) | 0.045 |
| Sustained ventricular arrhythmias | 12.70 (0.77, 24.63) | 0.037 |
| Reperfusion mode: successful thrombolysis | 56.53 (9.88, 103.19) | 0.018 |

Footnote: The coefficient (95% confidence intervals) indicates the magnitude and direction of the difference in the change in left ventricular end-diastolic volume (ml) for the patient characteristic (binary or continuous). For example, on average, the change in left ventricular end-diastolic volume (ml) at follow-up compared to baseline increases by 0.13 (0.01, 0.25) for each 1 ms increase in remote zone native T1 at baseline.

The clinical characteristics that were univariable predictors of change in left ventricular end-diastolic volume at follow-up that were also included in the multivariable model were age (p=0.923), body mass index (p=0.925), previous myocardial infarction (p=0.553), diabetes mellitus (p=0.273), previous percutaneous coronary intervention (p=0.974), cigarette smoking (p=0.357), history of angina (p=0.787), heart rate (p=0.484), systolic blood pressure on arrival in the cardiac catheterization laboratory (p=0.313), Killip class II vs. class I (reference category) (p=0.286), Killip class III vs. class I (reference category) (p=0.469), Killip class IV vs. class I (reference category) (p=0.263), symptom onset to reperfusion time (p=0.874), TIMI flow grade 2 vs. grade 0/1 (reference category) at initial angiography (p=0.194), TIMI flow grade 3 vs. grade 0/1 (reference category) at initial angiography (p=0.254), TIMI flow grade 2 grade 0/1 (reference category) at the end of PCI (p=0.771), TIMI flow grade 3 vs. grade 0/1 (reference category) at the end of PCI (p=0.519), ST segment resolution (none vs. complete (reference category), p=0.078; partial vs. complete (reference category), p=0.324), reperfusion mode (failed vs. primary PCI (reference category), p=0.188).

†When area-at-risk was included in place of infarct size, remote zone T1 was also a predictor of change in left ventricular end-diastolic volume at follow-up 0.13 (0.01, 0.24); p=0.031).

**Supplementary Table 6.** Clinical and angiographic characteristics of 151 STEMI patients who had CMR with evaluable T1 maps at baseline and NT-proBNP results at 6 months. The patients are grouped according to tertiles of remote zone native T1 values (ms) at baseline.

| Characteristics* |  | All patients | STEMI patients group,  tertile of remote zone native T1 at baseline | | |
| --- | --- | --- | --- | --- | --- |
|  |  |  | ≤ 951 ms | > 951 to ≤ 969 ms | > 969 ms |
|  |  | n=151 | n = 52 | n = 51 | n = 48 |
| Age, years |  | 59 (11) | 60 (10) | 59 (12) | 59 (11) |
| Male sex, n (%) |  | 121 (80) | 48 (92) | 40 (78) | 33 (69) |
| BMI, (kg/m^2^) |  | 29 (4) | 29 (4) | 29 (4) | 29 (5) |
| Hypertension, n (%) |  | 50 (33) | 12 (23) | 21 (42) | 17 (35) |
| Current smoking, n (%) |  | 84 (55) | 27 (52) | 29 (56) | 28 (68) |
| Hypercholesterolemia, n (%) |  | 40 (26) | 12 (23) | 15 (29) | 13 (27) |
| Diabetes mellitus‡, n (%) |  | 12 (8) | 4 (8) | 6 (12) | 2 (4) |
| Previous angina, n (%) |  | 16 (11) | 5 (10) | 4 (8) | 7 (15) |
| Previous myocardial infarction, n (%) |  | 11 (7) | 2 (4) | 3 (6) | 6 (12) |
| Previous PCI, n (%) |  | 8 (5) | 2 (4) | 2 (4) | 4 (8) |
| *Presenting characteristics* |  |  |  |  |  |
| Heart rate, bpm |  | 78 (16) | 75 (16) | 79 (16) | 78 (16) |
| Systolic blood pressure, mmHg |  | 139 (25) | 135 (25) | 143 (27) | 138 (24) |
| Diastolic blood pressure, mmHg |  | 81 (15) | 79 (11) | 84 (16) | 79 (17) |
| Time from symptom onset to reperfusion, min |  | 235 (212) | 216 (166) | 211 (212) | 282 (248) |
| Ventricular fibrillation†, n (%) |  | 11 (7) | 4 (8) | 4 (8) | 3 (6) |
| Heart failure Killip class, n (%) | I | 111 (74) | 40 (77) | 41 (81) | 30 (62) |
|  | II | 32 (21) | 10 (19) | 7 (14) | 15 (31) |
|  | III or IV | 8 (5) | 2 (4) | 3 (6) | 3 (6) |
| ECG |  |  |  |  |  |
| ST segment elevation resolution post PCI, n (%) |  |  |  |  |  |
| Complete, ≥70 % |  | 70 (46) | 32 (62) | 7 (14) | 8 (17) |
| Incomplete, 30% to < 70% |  | 57 (38) | 11 (21) | 22 (42) | 24 (50) |
| None, ≤30% |  | 24 (16) | 9 (17) | 22 (43) | 16 (33) |
| *Coronary angiography* |  |  |  |  |  |
| Reperfusion strategy, n (%) |  |  |  |  |  |
| Primary PCI |  | 143 (95) | 49 (94) | 47 (92) | 47 (98) |
| Rescue PCI (failed thrombolysis) |  | 7 (4) | 2 (4) | 4 (8) | 1 (2) |
| Successful thrombolysis |  | 1 (1) | 1 (2) | 0 (0) | 0 (0) |
| Number of diseased arteries¥, n (%) | 1 | 73 (48) | 27 (52) | 26 (51) | 20 (42) |
|  | 2 | 49 (32) | 18 (35) | 17 (33) | 14 (29) |
|  | 3 | 26 (17) | 7 (14) | 7 (14) | 12 (25) |
|  | Left main | 3 (2) | 0 (0) | 1 (2) | 2 (4) |
| Culprit artery, n (%) | Left anterior descending | 55 (36) | 16 (31) | 15 (29) | 24 (50) |
|  | Left circumflex | 28 (18) | 7 (14) | 12 (24) | 19 (19) |
|  | Right coronary | 68 (45) | 29 (56) | 24 (47) | 15 (31) |
| TIMI coronary flow grade pre-PCI, n (%) | 0/1 | 109 (72) | 32 (62) | 41 (80) | 36 (75) |
|  | 2 | 29 (19) | 13 (25) | 6 (12) | 10 (21) |
|  | 3 | 13 (9) | 7 (14) | 4 (8) | 2 (4) |
| TIMI coronary flow grade post-PCI, n (%) | 0/1 | 0 (0) | 0 (0) | 0 (0) | 0 (0) |
|  | 2 | 2 (1) | 1 (2) | 0 (0) | 1 (2) |
|  | 3 | 149 (99) | 51 (98) | 51 (100) | 47 (98) |
| *Initial blood results on admission* |  |  |  |  |  |
| C-reactive protein, (mg/L) | Median (IQR)  Range | 3.0 (2.0 - 7.0)  0 - 68.0 | 2.0 (1.0 – 4.5)  0 - 43.0 | 3.0 (2.0 - 7.0)  1.0 - 68.0 | 5.0 (2.5 – 9.0)  1.0 - 37.0 |
| Leucocyte cell count (x10^9^L) |  | 12.1 (3.2) | 11.0 (2.4) | 12.7 (3.4) | 12.8 (3.5) |
| Neutrophil count (x10^9^L) |  | 9.3 (3.0) | 8.2 (2.2) | 9.8 (3.1) | 10.1 (3.3) |
| Monocytes (x10^9^L) |  | 0.8 (0.3) | 0.8 (0.2) | 0.9 (0.3) | 0.8 (0.4) |

Footnote: TIMI = Thrombolysis in Myocardial Infarction grade, PCI = percutaneous coronary intervention. Killip classification of heart failure after acute myocardial infarction: class I - no heart failure, class II - pulmonary rales or crepitations, a third heart sound, and elevated jugular venous pressure, class III - acute pulmonary edema, class IV - cardiogenic shock. * Data are given as n (%) or mean (SD). ‡ Diabetes mellitus was defined as a history of diet-controlled or treated diabetes. † Successfully electrically cardioverted ventricular fibrillation at presentation or during emergency PCI procedure. ¥ Multivessel coronary artery disease was defined according to the number of stenoses of at least 50% of the reference vessel diameter, by visual assessment and whether or not there was left main stem involvement. P-values were obtained from one-way ANOVA or a Fisher test.

**Supplementary Table 7.** CMR findings at baseline (n=288) and at 6 months (n=267) in STEMI patients grouped according to tertiles of remote zone native T1 values (ms) at baseline.

| Characteristics* | All patients | STEMI patient group,  tertile of remote zone native T1 at baseline | | | P-value |
| --- | --- | --- | --- | --- | --- |
|  |  | ≤ 951 ms | > 951 to ≤ 969 ms | > 969 ms |  |
|  | n = 151 | n = 52 | n = 51 | n = 48 |  |
| *CMR findings 2 days post-MI (n=288)* |  |  |  |  |  |
| LV ejection fraction, % | 55 (10) | 56 (10) | 56 (9) | 53 (10) | 0.041 |
| LV end-diastolic volume, ml |  |  |  |  |  |
| Men | 160 (34) | 156 (30) | 154 (34) | 173 (35) | 0.017 |
| Women | 130 (22) | 135 (30) | 131 (24) | 127 (21) | 0.233 |
| LV end-systolic volume, ml |  |  |  |  |  |
| Men | 75 (25) | 69 (25) | 72 (23) | 87 (26) | <0.001 |
| Women | 56 (17) | 62 (23) | 58 (16) | 53 (17) | 0.476 |
| LV mass, g |  |  |  |  |  |
| Men | 146 (31) | 141 (30) | 143 (28) | 152 (33) | 0.054 |
| Women | 103 (22) | 112 (33) | 111 (20) | 94 (19) | 0.064 |
| *Edema and infarct characteristics* |  |  |  |  |  |
| Area at risk, % LV mass | 31 (12) | 28 (11) | 32 (12) | 33 (12) | 0.045 |
| Infarct size, % LV mass | 16 (13) | 13 (12) | 16 (13) | 21 (14) | 0.044 |
| Myocardial salvage, % of LV mass | 19 (9) | 19 (9) | 20 (9) | 18 (8) | 0.370 |
| Myocardial salvage index, % of LV mass | 65 (24) | 72 (25) | 64 (23) | 56 (23) | 0.013 |
| Late microvascular obstruction present, n (%) | 69 (46) | 18 (35) | 24 (47) | 27 (56) | 0.192 |
| Late microvascular obstruction, % LV mass | 2.6 (4.5) | 1.3 (3.3) | 3.0 (4.6) | 3.5 (5.1) | 0.021 |
| *Myocardial native T1 values* |  |  |  |  |  |
| T1 remote myocardium (all subjects), ms | 960 (23) | 936 (12) | 960 (6) | 986 (14) | <0.001 |
| Men, ms | 958 (23) | 935 (12) | 960 (6) | 987 (14) | <0.001 |
| Women, ms | 968 (21) | 934 (7) | 958 (6) | 984 (13) | <0.001 |
| T1 infarct zone, ms | 1096 (52) | 1076 (43) | 1100 (51) | 1101 (51) | <0.001 |
| T1 hypointense core present, n (%) | 81 (54) | 26 (50) | 26 (51) | 29 (60) | 0.483 |
| T1 hypointense infarct core, ms | 990 (53) | 991 (53) | 989 (53) | 991 (55) | 0.269 |
| *CMR findings 6 months post-MI (n=267)* |  |  |  |  |  |
| LV ejection fraction at 6 months, % | 62 (9) | 65 (7) | 63 (8) | 60 (11) | 0.010 |
| LV end-diastolic volume at 6 months, ml |  |  |  |  |  |
| Men | 168 (38) | 158 (34) | 164 (34) | 189 (42) | 0.007 |
| Women | 121 (21) | 112 (22) | 124 (18) | 122 (23) | 0.995 |
| LV end-systolic volume at 6 months, ml |  |  |  |  |  |
| Men | 66 (31) | 57 (22) | 64 (26) | 84 (39) | <0.001 |
| Women | 44 (15) | 39 (14) | 45 (14) | 44 (16) | 0.895 |

Footnote: * Data are given as n (%) or mean (SD). Abbreviations: LV = left ventricle, T1 = myocardial longitudinal relaxation time. Area-at-risk was measured with T2-mapping. P-values were obtained from one-way ANOVA, Kruskal-Wallis test or a Fisher test. CMR follow-up information was available in 267 STEMI patients (197 males and 70 females) with either LV dimensions or infarct size. Follow-up CMR results for changes in LV dimensions from baseline were available in 262 subjects (193 males and 69 females).

**Supplementary Table 8.**

Relationship between myocardial remote zone native T1 (ms) in 288 STEMI patients at baseline and subsequent MACE during longer term follow-up (median of 845 days follow-up (minimum - maximum post-discharge censor duration 598 - 1098 days). 39 patients experienced a MACE, out of whom 20 (6.9%) experienced a MACE post-discharge.

| Associations | Hazard ratio (95% CI) | p value |
| --- | --- | --- |
| *Univariable associations* |  |  |
| **Remote zone native T1, ms** | **1.016 (1.000, 1.032)** | **0.048** |
| LV end-diastolic volume at baseline, ml | 1.004 (0.992, 1.017) | 0.525 |
| Peak log C-reactive protein, (mg/L) | 1.425 (1.001, 2.029) | 0.049 |
| Change in lymphocyte count, x10^9^/L | 0.308 (0.093, 1.022) | 0.054 |
| Peak log basophil count, x10^9^/L | 0.621 (0.418, 0.922) | 0.018 |
| *Model A* | |  |
| LV end-diastolic volume at baseline, ml | 1.004 (0.991, 1.016) | 0.562 |
| **Remote zone native T1, ms** | **1.016 (1.000, 1.032)** | **0.045** |
| *Model B* |  |  |
| Peak lymphocyte count, x10^9^/L | 0.791 (0.449, 1.391) | 0.415 |
| Remote zone native T1, ms | 1.016 (1.001, 1.031) | 0.050 |
| *Model C* |  |  |
| Change in log C-reactive protein, (mg/L) | 1.046 (0.943, 1.161) | 0.392 |
| Remote zone native T1, ms | 1.016 (1.000, 1.032) | 0.045 |
| *Model D* |  |  |
| Change in leucocyte count, x10^9^/L | 1.059 (0.854, 1.313) | 0.603 |
| Remote zone native T1, ms | 1.017 (1.000, 1.033) | 0.043 |
| *Model E* |  |  |
| Change in neutrophil count, x10^9^/L | 1.009 (0.815, 1.248) | 0.936 |
| Remote zone native T1, ms | 1.017 (1.000, 1.033) | 0.041 |
| *Model F* |  |  |
| Change in monocyte count, x10^9^/L | 0.456 (0.057, 3.629) | 0.458 |
| Remote zone native T1, ms | 1.017 (1.001, 1.033) | 0.036 |
| *Model G* |  |  |
| Change in eosinophil count, x10^9^/L | 0.998 (0.771, 1.293) | 0.990 |
| Remote zone native T1, ms | 1.017 (1.001, 1.033) | 0.041 |
| *Model H* |  |  |
| Change in basophil count, x10^9^/L | 0.956 (0.793, 1.153) | 0.640 |
| Remote zone native T1, ms | 1.017 (1.001, 1.033) | 0.041 |
| *Model I* |  |  |
| Change in lymphocyte count, x10^9^/L | 0.324 (0.100, 1.052) | 0.061 |
| Remote zone native T1, ms | 1.016 (1.001, 1.032) | 0.046 |

Footnote: The index of native T1 in the infarct zone to native T1 in the remote zone was not associated with left ventricular or health outcomes in contrast to results with absolute native T1.

**Legend for Figure 1 (main text)**

**Figure 1.** Two patients with acute anterior STEMI treated by primary PCI and with the same standard anti-thrombotic therapies, including aspirin, clopidogrel and intravenous glycoprotein IIbIIIa inhibitor therapy with tirofiban. Each patient had TIMI grade 3 flow in the culprit left anterior descending coronary artery at the end of the procedure.

1. *Patient with high remote zone native T1*: The angiogram (left) revealed proximal occlusion of the left anterior descending artery (*yellow arrow*, TIMI grade 0 flow). CMR was performed 2 days later. Matched diastolic phase images obtained using late gadolinium enhancement imaging (*right*) revealed transmural infarction with microvascular obstruction (*yellow arrows*). T1 mapping (*middle right*) revealed a high remote zone native T1 value of 1049 ms. Acute infarct size revealed by late gadolinium enhancement imaging (*right*) was 48.1%. Microvascular obstruction depicted as the central dark zone within the infarct territory was 8·4% of LV mass. The LV ejection fraction and end-diastolic volume were 40·1% and 82.0 ml/m², respectively. Six month follow-up CMR revealed final infarct size was 39.2% of LV mass and significant adverse remodeling occurred with LV end-diastolic volume of 145.7 ml/m². This patient was subsequently hospitalized for new onset heart failure and had a defibrillator device implanted.
2. *Patient with average remote zone native T1 value:* The angiogram (*left*) revealed proximal occlusion of the left anterior descending artery (*yellow arrow,* TIMI grade 0 flow). CMR was performed two days later. T1 mapping (*middle right*) revealed a mean remote zone native T1 value of 962 ms. Acute infarct size revealed by late gadolinium enhancement (*right*) was 40.9%. Microvascular obstruction depicted as the central dark zone within the infarct territory was 5.4% of LV mass. The LV ejection fraction and end-systolic volume were 42·4% and 83.3 ml/m², respectively. The infarct size at 6 months revealed by contrast-enhanced CMR was 31.0% of LV mass and LV end-diastolic volume of 84.3 ml/m². This patient had an uncomplicated clinical course.

**Figure 2.** Flow diagram of the cohort study.

**Figure 3.** Remote zone native T1 (ms) at baseline was associated with NT-proBNP (median (IQR)) after 6 months. 151 STEMI patients had NT-proBNP results available at 6 months. According to tertile of remote zone native T1 two days post-MI (low vs. intermediate vs. upper), the NT-proBNP concentrations at 6 months were 105.00 (61.00, 179.00) pg/mL vs. 165.00 (77.75, 354.50) pg/mL vs. 233.00 (110.75, 498.50) pg/mL, respectively (p=0.002) (n=151).

**Supplementary Figure Legends**

**Figure 1.** Bland-Altman plot for inter-observer agreement of myocardial remote zone native T1 values obtained in 20 STEMI who underwent CMR 2 days post-MI.

**Figure 2.** Association between myocardial remote zone native T1 and change in left ventricular end-diastolic volume at 6 months (r=0.113, p=0.068).

**Supplementary Figure 1.**

**Supplementary Figure 2.**

**Remote zone native T1 (ms) disclosed by CMR in 267 STEMI patients 2 days post-MI and change in LV end-diastolic volume at 6 months.**
